# Pyrimethamine Restores KEAP1-Mediated Degradation of Select NRF2 Mutants in Esophageal Squamous Cell Carcinoma

**DOI:** 10.3390/cancers18091354

**Published:** 2026-04-24

**Authors:** Zhaohui Xiong, Chorlada Paiboonrungruang, Haining Wang, Boopathi Subramaniyan, Candice Bui-Linh, Yahui Li, Huan Li, Michael C. Wang, Francis Spitz, Xiaoxin Chen

**Affiliations:** 1 Coriell Institute for Medical Research, Camden, NJ 08103, USA; cpaiboonrungruang@coriell.org (C.P.); cbui-linh@coriell.org (C.B.-L.); 2 Insilico Medicine Canada Inc., Montreal, QC H3B 4W8, Canada; haining.wang@insilicomedicine.com; 3 Surgical Research Lab, Department of Surgery, Cooper University Health Care, Camden, NJ 08103, USA; subramaniyan-boopath@cooperhealth.edu (B.S.); li-yahui@cooperhealth.edu (Y.L.); li-huan@cooperhealth.edu (H.L.); wang-michael1@cooperhealth.edu (M.C.W.); spitz-francis@cooperhealth.edu (F.S.); 4 MD Anderson Cancer Center at Cooper, Camden, NJ 08103, USA

**Keywords:** esophageal squamous cell carcinoma, NRF2, KEAP1, pyrimethamine

## Abstract

**Background**: Esophageal squamous cell carcinoma (ESCC) remains a highly lethal malignancy with limited therapeutic options, in part due to frequent activation of nuclear factor erythroid 2-related factor 2 (NFE2L2 or NRF2). Gain-of-function mutations in NRF2 disrupt its negative regulation by Kelch-like ECH-associated protein 1 (KEAP1), resulting in sustained NRF2 signaling that promotes tumor growth and resistance to chemotherapy and radiation. We previously identified the FDA-approved drug pyrimethamine (PYR) as an NRF2 inhibitor and demonstrated that inhibition of dihydrofolate reductase (DHFR) represents the primary mechanism underlying its NRF2-suppressive activity, supporting its advancement into a Phase I window-of-opportunity clinical trial (NCT 05678348). Meanwhile, in NRF2^W24C^-KYSE70 and NRF2^D77V^-KYSE180 cells, PYR promoted NRF2^Mut^ ubiquitination and proteasomal degradation and shortened its half-life. This study aims to explore additional modes of action by which PYR inhibits NRF2. **Methods**: Cell cycle analysis was performed by flow cytometry. Cell proliferation, apoptosis and chemosensitivity were assessed by Live-Cell Analysis System, while radiosensitivity was evaluated using X-ray irradiation and the CellTiter-Glo assay. Molecular interactions between NRF2 and KEAP1 were examined through Co-IP and PLA, and the direct binding of PYR to KEAP1 was quantified using ITC and SPR. Molecular docking and dynamic simulations were employed to predict potential PYR-binding pockets within the Kelch domain. **Results**: Using genetically defined isogenic ESCC cell models, we show that activation of mutant NRF2 (NRF2^Mut^) or wild-type NRF2 (NRF2^WT^) produces distinct, context-dependent effects on squamous differentiation, proliferation, and therapeutic response. We further demonstrate that PYR restores sensitivity to chemotherapy and ionizing radiation in NRF2^Mut^ ESCC cells. Mechanistically, short-term PYR treatment promotes KEAP1-dependent proteasome-mediated degradation of NRF2^W24C^. Biochemical and biophysical assays indicate that PYR enhances the interaction between KEAP1 and NRF2^W24C^ in a manner associated with KEAP1-dependent proteasomal degradation. Computational modeling further suggests that PYR may engage a pocket within the Kelch domain to facilitate the NRF2^W24C^-KEAP1 interaction. **Conclusions**: These findings show that PYR functionally restores KEAP1-mediated NRF2 degradation of select NRF2^Mut^ through a glue-like effect and overcomes therapy resistance in ESCC. Although the proposed glue-like mechanism remains hypothetical, this work supports further investigation into the NRF2–KEAP1 interaction and may inform the development of KEAP1-targeted strategies for NRF2^Mut^ cancers, including ESCC.

## Simple Summary

Esophageal cancer is a deadly disease that often becomes resistant to treatment because cancer cells activate protective pathways that help them survive. In this study, we aimed to understand how a commonly used drug, pyrimethamine, could help overcome this resistance. We found that certain genetic changes in a key protective protein make cancer cells grow faster and resist chemotherapy and radiation. Importantly, pyrimethamine was able to restore treatment sensitivity in these cancer cells by helping another protein mark the harmful form of this protective protein for destruction. This effect appears to involve a “glue-like” action that brings the two proteins (NRF2 and KEAP1) closer together, although the exact details still need further study. Our findings suggest that this drug, which is already approved for other uses, could be repurposed to treat a subset of esophageal cancers with these specific genetic changes. More broadly, this work highlights a potential new strategy for designing treatments that selectively target cancer-causing protein variants while sparing normal cells, which could lead to safer and more effective therapies in the future.

## 1. Introduction

Esophageal squamous cell carcinoma (ESCC) is a highly aggressive malignancy and ranks among the most lethal cancers of the gastrointestinal tract worldwide. Despite advances in surgery, radiation, and chemotherapy, the five-year survival rate for patients with advanced-stage ESCC remains dismal [1,2]. One of the factors contributing to therapeutic resistance and poor prognosis in ESCC is hyperactivation of nuclear factor erythroid 2-related factor 2 (NFE2L2 or NRF2), a master transcription factor that regulates cellular defense mechanisms against oxidative stress, xenobiotics, and metabolic injury. A total of 24.5% of ESCC was classified as the “NRF2 oncogenic activation subtype” based on multi-omics data [3,4]. Under physiological conditions, NRF2 activity is tightly controlled by its negative regulator, Kelch-like ECH-associated protein 1 (KEAP1), which targets NRF2 for ubiquitination and subsequent proteasomal degradation. This regulation is mediated through direct binding of two conserved motifs within NRF2—the high-affinity ETGE motif and the lower-affinity DLG motif—to the Kelch domain of KEAP1, enabling proper positioning of NRF2 for ubiquitination by the CUL3-based E3 ligase complex [5]. However, in approximately 10–22% of human ESCC cases, this regulatory axis is disrupted by gain-of-function mutations in NRF2 (NRF2^Mut^) or loss-of-function mutations in KEAP1, leading to persistent NRF2 stabilization and nuclear accumulation [6,7,8]. Mutations in KEAP1 and CUL3 are present but less common than those in NRF2. According to two sequencing studies on 227 cases of ESCC, NRF2, KEAP1, and CUL3 mutation took place in 5.28%, 2.8%, and 0.9% cases, respectively. In an integrated dataset that consists of 1930 ESCC genomes from 33 datasets, NRF2, KEAP1, and CUL3 mutation took place in 7.62%, 2.75% and 2.49% cases, respectively. Depending on the patient population, the frequency of NRF2 and KEAP1 mutations can be as high as 35.9% and 4.1%, respectively [8]. Although NRF2 plays a protective role in normal tissues by mitigating oxidative stress, its sustained activation in cancer cells confers a survival advantage, enhances proliferation, rewires metabolic pathways, and drives resistance to chemotherapy, radiation, and immunotherapy [5,6,9,10]. These observations underscore NRF2 as a compelling therapeutic target, especially in cancers like ESCC where mutations affecting the NRF2-KEAP1 interaction are common.

Despite its importance, targeting NRF2 pharmacologically has proven challenging, as it is considered an ‘undruggable’ target due to its function as a transcription factor lacking classical enzymatic pockets or well-defined ligand-binding sites [11,12]. Nonetheless, significant progress has been made in recent years. A notable example is VVD-065, a molecular glue that specifically and covalently engages Cys^151^ on the BTB domain of KEAP1, which in turn promotes KEAP1-CUL3 interaction, leading to enhancement of degradation of both wild-type NRF2 (NRF2^WT^) and NRF2^Mut^. VVD-065 reduces NRF2 levels only in settings where KEAP1 and NRF2 are able to physically interact with each other. This mechanistic constraint allows for coverage of NRF2 “DLG” region mutations, a subset of KEAP1 mutations known as anchor mutations, and wild-type KEAP1-NRF2 [13]. A related compound, VVD-130037, which employs the same molecular glue mechanism, is currently undergoing Phase 1 clinical trial (NCT05954312).

Another promising agent is pyrimethamine (PYR), an FDA-approved drug long used to treat malaria and toxoplasmosis by inhibiting dihydrofolate reductase (DHFR) [14]. We identified PYR as a novel NRF2 inhibitor through small-molecule screening and validated its efficacy both in vitro and in vivo [15]. PYR was found to suppress NRF2 expression and attenuate NRF2^E79Q^-driven esophageal hyperplasia in a mouse model. Importantly, it achieved these effects at clinically relevant and safe doses, making it an ideal candidate for drug repurpose. In support of its translational potential, PYR has progressed to a Phase 1 clinical trial (NCT05678348). Genetic, pharmacologic, and metabolic epistasis studies demonstrated that DHFR inhibition was essential for the NRF2-inhibitory activity of PYR and its structural analog WCDD115. Proteomic analyses showed that PYR shares a molecular signature with methotrexate (MTX), a classical DHFR inhibitor [16]. On the other hand, in NRF2^W24C^-KYSE70 and NRF2^D77V^-KYSE180 cells, PYR promoted NRF2^Mut^ ubiquitination and proteasomal degradation and shortened its half-life, suggesting an additional mode of action [15]. Interestingly, similar protein degradation effect of PYR was observed with the exon 2-depleted splice variant of aminoacyl-tRNA synthetase-interacting multifunctional protein 2 (AIMP2-DX2) in lung cancer [17]. These findings underscore the potential of PYR as a dual-function NRF2 inhibitor, acting through DHFR inhibition and direct NRF2 degradation. While PYR is known to inhibit NRF2 primarily through DHFR inhibition, the mechanism by which it promotes degradation of NRF2^W24C^ and NRF2^D77V^ remains unclear, as does whether PYR exerts similar effects on other NRF2^Mut^ and NRF2^WT^. The current study aims to further dissect PYR’s mechanisms of action, which may inform the development of more potent and specific inhibitors for NRF2^Mut^ malignancies including ESCC.

## 2. Materials and Methods

### 2.1. Cell Culture and Chemicals

Human ESCC cells, KYSE70 (NRF2^W24C^), KYSE180 (NRF2^D77V^), and KYSE450 (NRF2^WT^) were purchased from DSMZ (Braunschweig, Germany). TE14 (NRF2^D29H^) and OE21 (NRF2^G81S^) cells were obtained from Columbia University (New York, NY, USA) and Sigma-Aldrich (St. Louis, MO, USA), respectively. These cell lines represent diverse tumor phenotypes: KYSE70 is poorly differentiated and more aggressive, with higher invasive potential but slower proliferation. KYSE450 is more differentiated and exhibits faster growth. TE14 is moderately differentiated with intermediate growth and invasion characteristics. OE21 is also relatively well differentiated with strong epithelial features and robust growth. All cells were cultured in Gibco RPMI1640 (ThermoFisher, Waltham, MA, USA) supplemented with 10% FBS and 1% antibiotics (penicillin/streptomycin) at 37 °C under humidified conditions with 5% CO_2_. PYR, MTX, 5-fluorouracil (5-FU) and cisplatin were purchased from Sigma-Aldrich.

NRF2^null^-KYSE70, KEAP1^null^-KYSE70, and KEAP1^null^-KYSE450 cells were generated by using CRISPR-Cas9-mediated genome editing (Figures S1 and S2). Briefly, sgRNAs were designed to target coding exons to ensure biallelic loss-of-function mutations. For KEAP1^null^-KYSE450 cells, the sgRNA sequence 5′-GAAGGUGCGGUUGCCAUGCU-3′ was designed to target exon 2 of the *KEAP1* gene. For KEAP1^null^-KYSE70 cells, the sgRNA sequence 5′-CUGCUUGGUAUGAUCCUCCA-3′ targets exon 2 of the *KEAP1* gene. For NRF2^null^-KYSE70 cells, the sgRNA sequence 5′-UAUUUGACUUCAGUCAGCGA-3′ targets exon 2 of the *NFE2L2* gene. Following single-cell cloning, knockout was confirmed by Sanger sequencing and further validated at the protein level by Western blotting. To minimize potential off-target effects, sgRNAs were selected using established CRISPR design tools with high predicted on-target activity and low off-target potential.

### 2.2. RT-PCR

Total RNA was isolated using Qiagen RNeasy Mini Kit (Germantown, MD, USA) and reverse-transcribed using the Advantage RT-for-PCR kit (Clontech, Mountain View, CA, USA), according to the manufacturer’s instructions. mRNA transcripts were detected with the following primer pair: forward (5′-TCATGATGGACTTGGAGCTG-3′) and reverse (5′-GCAATGAAGACTGGGCTCTC-3′). This primer pair was expected to amplify all *NFE2L2* transcripts including transcript 1 (475 bp), transcript 6 (385 bp), and transcript 7 (256 bp) [18].

### 2.3. Western Blotting and Co-Immunoprecipitation (Co-IP)

The whole cell lysates were made using RIPA lysis buffer supplemented with a protease inhibitor cocktail (ThermoFisher). Proteins were separated by SDS-PAGE and transferred to nitrocellulose membranes. Membranes were blocked and then incubated with a primary antibody overnight at 4 °C (Table S1). Chemiluminescence was detected using iBright FL1500 (ThermoFisher) and followed by quantification using ImageJ 1.54d. To analyze NRF2-KEAP1 binding, cells were treated with PYR or MTX for 2 h before being harvested in RIPA buffer. The cell lysate was pre-cleaned with protein G agarose beads (Roche Diagnostic, Basel, Switzerland) for 1 h at 4 °C. Samples were incubated with 1 µg anti-NRF2 or anti-KEAP1 antibody overnight at 4 °C with rotation, and then 25 µL protein G agarose beads was added for 1.5 h at 4 °C. Immunoprecipitants were washed and boiled in 2× SDS loading buffer at 95 °C for 5 min, and analyzed by Western blotting.

### 2.4. Cell Cycle Analysis

Cells were harvested by trypsinization, washed with PBS, and fixed in 70% ethanol. Fixed cells were stored at −20 °C overnight. Cells were washed and resuspended in PBS containing RNase A, filtered, and incubated with propidium iodide (50 µg/mL) for 1 h at 4 °C in the dark. Cell cycle analysis was performed using the BD FACSymphony™ A3 Cell Analyzer (BD Biosciences, Milpitas, CA, USA) and FlowJo™ v10 software (BD Biosciences).

### 2.5. Proliferation Assay

Cells were seeded in 96-well plates and incubated with NucRed™ Live 647 ReadyProbes™ Reagent (ThermoFisher) to label nuclei. Plates were imaged every 4 h over a 72 h period using the Incucyte^®^ SX5 Live-Cell Analysis System (Sartorius, Göttingen, Germany). Fluorescent images were analyzed using the Incucyte 2023A Rev2, and proliferation curves were generated using GraphPad Prism 10 (GraphPad Software, La Jolla, CA, USA).

### 2.6. Apoptosis Assay

Apoptosis was assessed using the CellEvent™ Caspase-3/7 Green Detection Reagent (ThermoFisher). Cells were seeded in 96-well plates and treated as indicated. Caspase-3/7 reagent was added according to the manufacturer’s instructions. Plates were imaged every 4 h for 72 h using the ImageXpress Pico Automated Cell Imaging System (Molecular Devices, San Jose, CA, USA). Green fluorescence indicating caspase-3/7 activation was quantified using the system’s integrated analysis software.

### 2.7. Chemosensitivity Assay

To evaluate the drug effects on cell viability, real-time imaging was performed using the IncuCyte platform. KYSE70 and NRF2^null^-KYSE70 cells were seeded in 96-well plates at a density of 1000 cells per well and incubated overnight to allow for cell adherence. Cells were pretreated with 10 µM PYR for 48 h. Subsequently, either 5-FU or cisplatin was administered to assess differential chemosensitivity. 5-FU was serially diluted in a 1:2 ratio from an initial concentration of 1000 µM, yielding a total of 11 concentrations plus a vehicle control (0 µM). Cisplatin was prepared using the same dilution scheme starting from 240 µM. To facilitate real-time monitoring of cell viability, NucRed™ Live 647 ReadyProbes™ Reagent (ThermoFisher) was utilized to stain nuclei, while YOYO-1 iodide (ThermoFisher) was employed as a cell-impermeant dye to selectively identify dead cells. Following treatment and dye addition, plates were transferred to the Incucyte platform. Phase-contrast and fluorescence images were acquired at 6 h intervals over a period of 48 h. Cell confluence and fluorescence signals were quantified using the Incucyte integrated analysis software.

### 2.8. Radiosensitivity Assays

Both 2D and 3D culture were used to test radiosensitivity. While 2D culture provides a standardized measure of intrinsic cellular radiosensitivity, 3D culture more faithfully reflects the in vivo tumor microenvironment, including hypoxia and cell–cell interactions that modulate radiation response. To determine the effects of NRF2^WT^ and NRF2^W24C^ on radiosensitivity of isogenic cells, cells were seeded into a 3D pillar plate format on Day 0 using the MBD ASFA^®^ Spotter (Medical & Bio Decision, Suwon-si, Republic of Korea), which enables precise, non-contact dispensing of cell-laden hydrogel droplets. Each spot contained 1000 cells embedded in a 3D matrix to support spheroid formation. On Day 1 and Day 4, the cultured cells were exposed to varying doses of ionizing radiation to assess radiosensitivity. On Day 7, cell viability was evaluated using the CellTiter-Glo^®^ 3D Cell Viability Assay (Promega, Madison, WI, USA) following the manufacturer’s instructions.

The effect of PYR in combination with ionizing radiation on cell viability was assessed in 2D-cultured cells. Cells were seeded into 96-well plates at a density of 2500 cells per well and incubated overnight to allow for adherence. Cells were pretreated with 10 µM PYR for 48 h, followed by exposure to graded doses of X-ray irradiation (0, 2, 4, 6, 8, and 10 Gy) using an irradiator (RS2000; RadSource, Brentwood, TN, USA). After irradiation, cells were incubated for an additional 72 h under standard culture conditions. Cell viability was assessed using the CellTiter-Glo^®^ Luminescent Cell Viability Assay following the manufacturer’s instructions. Luminescence was measured using a microplate reader SpectraMax^®^ iD5 (Molecular Devices, San Jose, CA, USA), and viability was normalized to the non-irradiated control.

### 2.9. Metabolomic Profiling

KYSE70 and NRF2^null^-KYSE70 cell pellets (*n* = 3 per cell type) were analyzed by Metabolon (Durham, NC, USA) with ultra-high-performance liquid chromatography–tandem mass spectroscopy. Raw data were extracted, peak-identified and QC processed by Metabolon.

### 2.10. RNAseq

RNAseq was performed by Admera Health (South Plainfield, NJ, USA). Total RNA was used as input material for the RNA sample preparations (*n* = 3 per cell type). Sequencing libraries were generated using NEBNext Ultra TM RNA Library Prep Kit for Illumina (NEB, Ipswich, MA, USA) following the manufacturer’s recommendations and index codes were added to attribute sequences to each sample. After library preparation, the samples were sequenced (150 bp) according to manufacturer specifications. The quality-filtered reads were aligned with STAR (version 2.6.90c) to the human reference genome (hg38) with its respective RefSeq annotation, and the expression levels of genes were obtained with Feature Counts (version 1.5.1). DESeq2 (version 1.34.1) was used to identify the differentially expressed genes (DEGs). Data matrices were normalized and the DEGs were reported according to the fold change cut-off and corrected modeling *p*-values. The Benjamini–Hochberg method was applied to calculate adjusted *p*-values. Genes were considered significantly differentially expressed with an adjusted *p*-value < 0.05 and log2FC cutoff of 1. Gene set enrichment analysis was conducted to evaluate the differential enrichment of gene sets including “NRF2 target gene set” [19]. The raw data has been deposited in NCBI Gene Expression Omnibus (GEO) at GSE 299159 and GSE 299160.

### 2.11. Proximity Ligation Assay (PLA)

The Duolink In Situ Red Fluorescent kit (Sigma-Aldrich) was employed to investigate the endogenous interaction between NRF2 and KEAP1 in human ESCC cells. The PLA was conducted following the manufacturer’s instructions. PLA with anti-NRF2 and anti-KEAP1 alone served as negative controls. A total of 1 × 10^6^ cells were plated on poly L-lysine-coated coverslips and allowed to adhere for 24 h. The cells were treated with PYR for 4 h and fixed using 4% paraformaldehyde and permeabilized with 0.1% Triton X-100 before being incubated with the antibodies. The nuclei were counterstained with DRAQ5 (ThermoFisher). Images were captured with an Olympus FV3000 confocal microscope (Olympus, Tokyo, Japan) and processed with ImageJ 1.54dX. PLA signals in a minimum of 100 cells were counted using ImageJ.

### 2.12. Isothermal Titration Calorimetry (ITC)

To test the interaction between PYR and KEAP1, ITC assay was performed by Creative Biolabs (Shirley, NY, USA). A total of 4 μL aliquots of 100 μM PYR was injected from a syringe into the sample cell containing 200 μL of human recombinant KEAP1 (Gln^2^-Cys^624^, MedChemExpress, Monmouth Junction, NJ, USA). Each experiment was accompanied by the corresponding control experiment in which 4 μL aliquots of 100 μM PYR were injected into a buffer alone. The delay between injections was 150 s. Each injection generated a heat burst curve. Calorimetric titrations were performed at 25 °C with a speed of 250 rpm.

### 2.13. Surface Plasmon Resonance (SPR)

A Kelch fragment (Ala^321^-Glu^611^ of human KEAP1 protein, MW 34,038.03 Da) was expressed in vitro, purified, biotinylated, and validated by LC-MS. DLG^WT^ (^17^MDLIDILWRQDIDLGVSREVFDFS^40^) and DLG^W24C^ (^17^MDLIDILCRQDIDLGVSREVFDFS^40^) were synthesized. SPR analysis was performed using Biacore S200 (Cytiva, Marlborough, MA, USA) equipped with a streptavidin sensor chip. Biotinylated Kelch (4.05 mg/mL) was immobilized on flow cells, respectively, at a concentration of 10 μg/mL, with a flow rate of 5 μL/min for 44 or 49 s. Analytes (DLG^WT^ and DLG^W24C^) were prepared as 2-fold serial dilutions ranging from 200 μM to 0.39 μM, while PYR was diluted from 500 μM to 0.97 μM. To test the effects of PYR on the DLG–Kelch interaction, PYR was mixed in the analyte together with DLG peptides. Each analyte was injected at a flow rate of 30 μL/min with an association phase of 90 s and a dissociation phase of 180 s. The sample compartment was maintained at 25 °C, and the analysis was conducted at 15 °C. Non-specific binding was minimized by using a reference channel, applying surface blocking, and optimizing buffer conditions (including detergent and salt). Mass transport effects were reduced by employing appropriate flow rates, maintaining low ligand density, and verifying that kinetic parameters were independent of flow rate.

### 2.14. Molecular Docking

DiffDock was used to explore hypothetical PYR-binding sites on KEAP1. We further generated NRF2 structure with AlphaFold 2 (DeepMind, London, UK) and then docked PYR on DLG:KEAP1 (PDB: 3WN7) using MOE (Chemical Computing Group, Montreal, QC, Canada). Rigid docking with the Amber10:EHT force field was employed to generate the docking models using the following settings: placement (Triangle Matcher), refinement (Induced Fit), and scoring (London dG (retained 300 poses) followed by GBVI/WSA dG (top 5 poses). Binding energy was obtained using the Molecular Contact Surface Analysis script implemented in MOE, which estimates the interaction energy based on contact surface parameters. For protein–protein docking, we used the DLG^WT^ peptide (residues 17–40, sequence MDLIDILCRQDIDLGVSREVFDFS) and AlphaFold-predicted DLG^W24C^ peptide for docking into KEAP1 using 3WN7 as the structural template. Regarding the BTB domain, the candidate site was chosen based on literature reports of VVD-065, a covalent binder of KEAP1 in this domain [13]. Interaction between KEAP1 and three NRF2 activators which disrupts NRF2-KEAP1 interaction, PRL-295 [20], KI696 [21], and Compound 4 [22], were visualized to demonstrate the differences between PYR and these activators.

Chemistry42 (Insilico Medicine, Montreal, QC, Canada) [23] was also used to explore the PYR-binding pocket on KEAP1, with the conformer count set to 300 to increase conformational sampling. PYR was docked into the Kelch domain using the Chemistry42 Virtual Screening module. Docking was first performed without any constraints, followed by a second round in which a mandatory point (MP) was placed on Arg^415^. Protein–ligand interaction scores were calculated for all docking poses as an initial estimate of binding affinity. Both the unconstrained and MP-guided poses were further evaluated using MDflow molecular dynamics simulations to assess stability and determine the most plausible binding mode.

### 2.15. Statistical Analysis

All experiments were duplicated or triplicated to ensure reproducibility of the data. GraphPad Prism 10 (GraphPad Software) was used, as well as two-way ANOVA and Student’s *t*-test, with the statistical significance level set at 0.05. Drug response data were processed to generate dose–response curves and to calculate IC_50_ using GraphPad Prism. Statistical comparisons of drug response were performed using two-way ANOVA. For metabolomic profiling, refined data were analyzed by two-way ANOVA and plotted with GraphPad Prism. To demonstrate the synergistic effects, combination index (CI) analysis was performed using the CompuSyn software version 1.0 (CompuSyn Inc., Paramus, NJ, USA). This program defines synergism as an effect that exceeds the expected additive effect with a CI < 1.

## 3. Results

### 3.1. Establishment of Isogenic ESCC Cells with NRF2 or KEAP1 Deficiency

**Figure 1:**
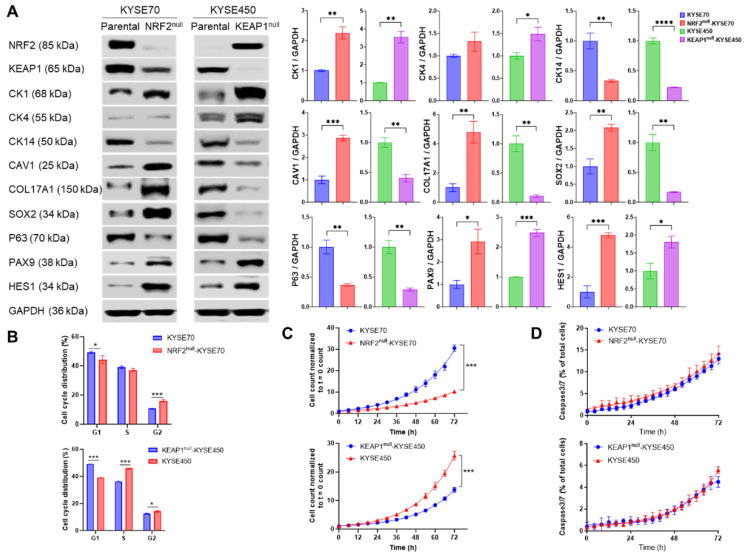
NRF2 differentially regulates gene expression and cellular behavior in ESCC cells. Isogenic NRF2^null^-KYSE70 cells were compared with NRF2^W24C^-KYSE70 cells, and KEAP1^null^-KYSE450 cells were compared with NRF2^WT^-KYSE450 cells. (**A**) Expression of squamous differentiation and basal cell markers assessed by Western blotting. (**B**) Cell-cycle distribution determined by flow cytometry. (**C**) Cell proliferation measured by IncuCyte live-cell imaging. (**D**) Apoptosis quantified by IncuCyte analysis. Data are shown as mean ± SD. * *p* < 0.05, ** *p* < 0.01, *** *p* < 0.001, **** *p* < 0.0001. Original western blots are presented in File S1.

To investigate the molecular and phenotypic consequences of NRF2 activation in ESCC, we generated isogenic human ESCC cell lines using CRISPR-Cas9-mediated genome editing. Monoclonal NRF2^null^-KYSE70, KEAP1^null^-KYSE70, and KEAP1^null^-KYSE450 cells were established and compared with their respective parental lines, NRF2^W24C^-KYSE70 and NRF2^WT^-KYSE450. These isogenic cell lines were validated by Western blotting, Sanger sequencing, RNA sequencing, and untargeted metabolomics (Figures S1, S2 and Figure 1). RNA-seq analysis confirmed that the “human ESCC NRF2 target gene set” [19] was significantly enriched in NRF2^W24C^-KYSE70 and KEAP1^null^-KYSE450 cells compared with their corresponding isogenic controls (Excel S1 and S2). Consistent with NRF2’s established role in metabolic regulation, metabolomic profiling revealed increased glutathione metabolism, glycolysis, gluconeogenesis, pyruvate metabolism, and phospholipid-related pathways in NRF2^W24C^-KYSE70 cells relative to NRF2^null^-KYSE70 cells (Excel S3). NRF2^null^-KYSE70 cells exhibited smaller cell size and slower growth compared to the parental cells whereas KEAP1^null^-KYSE450 cells and KEAP1^null^-KYSE70 cells showed no observable morphological differences from their respective parental cells under microscopic examination.

A faint, lower-molecular-weight NRF2 band was detected by Western blotting in NRF2^null^-KYSE70 cells (Figure 1A). Given that human *NRF2* mRNA has eight transcript variants [24], RT-PCR analysis revealed expression of transcript variants 6 and 7, encoding protein isoforms approximately 3 kDa and 8 kDa smaller than the predominant isoform, respectively (Figure S1C). Based on molecular weight, the residual NRF2 signal is most consistent with protein isoform 5.

### 3.2. NRF2 Activation Modulates Cellular Behaviors, Chemosensitivity and Radiosensitivity of Human ESCC Cells

RNAseq analysis revealed that both the “basal layer gene set” and “P63 target gene set” were enriched in KYSE450 cells in comparison to KEAP1^null^-KYSE450 cells, indicating that NRF2^WT^ overexpression promoted squamous differentiation (Excels S1 and S2). Consistent with the transcriptomic data, Western blotting demonstrated that NRF2^WT^ overexpression upregulated squamous differentiation markers (CK1, CK4) and NOTCH target genes (PAX9, HES1), while downregulating basal cell markers (CK14, CAV1, COL17A1, SOX2, P63) (Figure 1A). In contrast, NRF2^W24C^ expression exerted differential effects: it downregulated squamous differentiation markers (CK1) and NOTCH target genes (PAX9, HES1) but had a mixed effect on basal cell markers—downregulating CAV1, COL17A1, and SOX2, while upregulating CK14 and P63. These results suggest that NRF2^W24C^ may have distinct transcriptional outputs from NRF2^WT^, particularly in its regulation of squamous differentiation.

Flow cytometry analysis revealed that NRF2 activation altered cell cycle distribution in both KYSE70 and KYSE450 cells (Figure 1B). Live-cell imaging demonstrated that NRF2^W24C^ expression enhanced cell proliferation in KYSE70 cells, whereas NRF2^WT^ overexpression inhibited proliferation in KYSE450 cells (Figure 1C). Neither NRF2^WT^ nor NRF2^W24C^ significantly affected apoptosis in either cell line (Figure 1D). Together, these results indicate that NRF2^WT^ and NRF2^Mut^ exert distinct effects on cellular behavior in ESCC.

**Figure 2:**
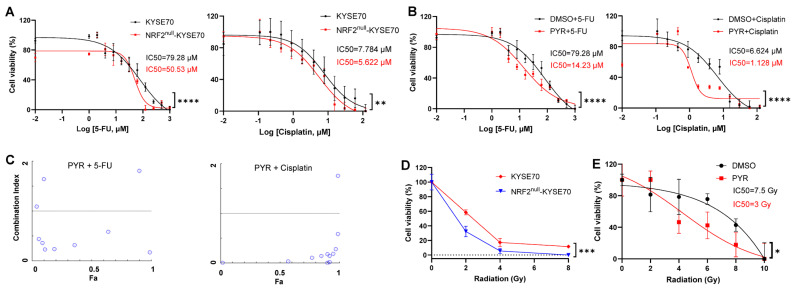
NRF2 activation reduces chemosensitivity and radiosensitivity, which is restored by PYR. (**A**) Cell viability of NRF2^W24C^-KYSE70 and NRF2^null^-KYSE70 cells following treatment with 5-FU or cisplatin. (**B**) Cell viability of NRF2^W24C^-KYSE70 cells following co-treatment with PYR (10 μM) and 5-FU or cisplatin. (**C**) CI analysis of PYR with 5-FU or cisplatin in NRF2^W24C^-KYSE70 cells. The dotted lines represent the expected additive effect (CI = 1) and the circles represent CI value for combination of drug dose. (**D**) Cell viability of NRF2^W24C^-KYSE70 and NRF2^null^-KYSE70 cells in 3D culture following radiation exposure. (**E**) Cell viability of NRF2^W24C^-KYSE70 cells treated with PYR (10 μM) and radiation in 2D culture. Data are shown as mean ± SD. * *p* < 0.05, ** *p* < 0.01, *** *p* < 0.001, **** *p* < 0.0001.

To assess the impact of NRF2 activation on chemosensitivity, we treated isogenic NRF2^null^-KYSE70 and NRF2^W24C^-KYSE70 cells with 5-FU or cisplatin for 48 h. As expected, 5-FU markedly reduced the viability of NRF2^null^-KYSE70 cells (IC_50_ = 50.53 µM), in comparison to NRF2^W24C^-KYSE70 cells (IC_50_ = 79.28 µM). A similar trend was observed with cisplatin, supporting a role for NRF2^W24C^ in promoting chemoresistance (Figure 2A).

Co-treatment with PYR significantly enhanced the cytotoxic effects of 5-FU and cisplatin in NRF2^W24C^-KYSE70 cells, reducing IC_50_ values to 14.23 µM and 1.128 µM, respectively (Figure 2B). Similar chemosensitizing effects were observed in NRF2^D77V^-KYSE180 cells (Figure S3A). Combination index analysis demonstrated synergistic interactions between PYR and both chemotherapeutic agents (Figure 2C). NRF2^null^-KYSE70 cells exhibited increased radiosensitivity in 3D culture (Figure 2D), whereas KEAP1^null^-KYSE450 cells displayed enhanced radioresistance (Figure S3B). Notably, PYR significantly sensitized NRF2^W24C^-KYSE70 cells to ionizing radiation in 2D culture, reducing the IC_50_ from 7.5 Gy to 3 Gy (Figure 2E). Collectively, these findings indicate that NRF2 activation confers resistance to chemotherapy and radiation, whereas pharmacologic inhibition with PYR restores therapeutic sensitivity. While the precise mechanisms underlying PYR-mediated radiosensitization were not dissected here, these effects are consistent with suppression of NRF2-dependent cytoprotective programs.

### 3.3. PYR Promotes KEAP1-Dependent Degradation of NRF2^W24C^

**Figure 3:**
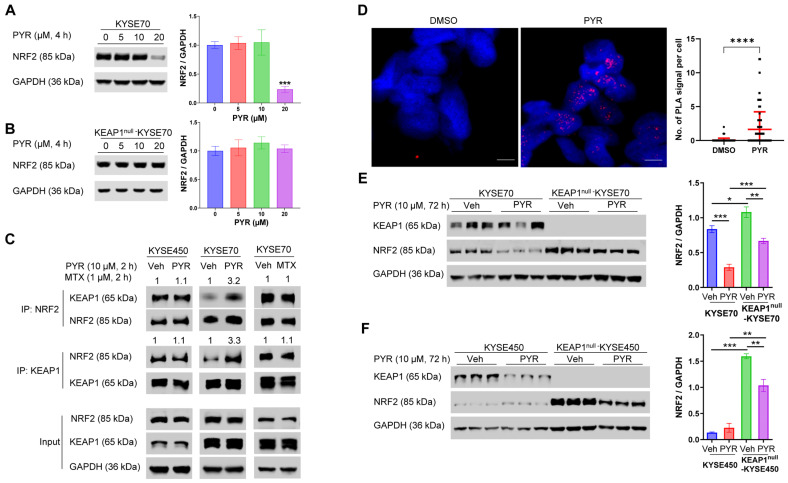
PYR enhances KEAP1-dependent degradation of NRF2^W24C^. (**A**) Dose-dependent reduction in NRF2^W24C^ protein levels in KYSE70 cells following 4 h PYR treatment. (**B**) Lack of NRF2^W24C^ reduction in KEAP1^null^-KYSE70 cells following 4 h PYR treatment. (**C**) Co-IP analysis showing increased association between NRF2^W24C^ and KEAP1 following PYR treatment (10 μM, 2 h), but not between NRF2^WT^ and KEAP1. MTX (1 μM, 2 h) did not alter NRF2^W24C^-KEAP1 association. (**D**) PLA showing increased NRF2^W24C^-KEAP1 interactions following PYR treatment (10 μM, 4 h). Red dots represent PLA signal. (**E**,**F**) NRF2 protein levels following 72 h PYR treatment (10 μM) in KEAP1^null^-KYSE70 and KEAP1^null^-KYSE450 cells. Data are shown as mean ± SD. * *p* < 0.05, ** *p* < 0.01, *** *p* < 0.001, **** *p* < 0.0001. Original western blots are presented in File S1.

Our previous study has shown that PYR reduced NRF2 half-life by promoting its ubiquitination and degradation in NRF2^W24C^-KYSE70 and NRF2^D77V^-KYSE180 cells, but not in NRF2^WT^-KYSE450 cells [15]. Similarly, PYR has been reported to promote ubiquitination and degradation of AIMP2-DX2 in lung cancer cells [17]. In agreement with these observations, PYR treatment for 4 h was found to inhibit NRF2^W24C^ expression in KYSE70 cells in a dose-dependent manner (Figure 3A). This inhibitory effect on NRF2^W24C^ was dependent on the presence of KEAP1, as PYR did not suppress NRF2^W24C^ expression in KEAP1^null^-KYSE70 cells (Figure 3B). PYR also significantly reduced NRF2 expression in NRF2^D77V^-KYSE180 cells, but had no such effect on NRF2^WT^-KYSE450, NRF2^D29H^-TE14, or NRF2^G81S^-OE21 cells (Figure S4A–D). Inhibition of the proteasome with MG132 abolished PYR-induced NRF2^W24C^ degradation, indicating a proteasome-dependent mechanism (Figure S4E).

Co-IP experiment revealed that PYR (10 μM, 2 h) enhanced the interaction between NRF2^W24C^ and KEAP1, but not between NRF2^WT^ and KEAP1 (Figure 3C). In contrast, MTX, another DHFR inhibitor, did not affect NRF2^W24C^-KEAP1 association, suggesting that this effect is independent of DHFR inhibition. PLA analysis further showed increased NRF2^W24C^-KEAP1 interactions following PYR treatment, with signals predominantly localized to the nucleus (Figure 3D). These findings are consistent with prior reports describing nuclear KEAP1–NRF2 complex formation followed by nuclear export and proteasomal degradation of NRF2 [25].

At longer treatment durations (72 h), PYR reduced NRF2 levels even in KEAP1^null^ cells, although to a lesser extent than in KEAP1-proficient cells (Figure 3E,F), suggesting an additional KEAP1-independent component of NRF2 suppression, likely mediated by DHFR inhibition [16].

### 3.4. Biophysical and Computational Analyses Suggest Enhancement of NRF2^W24C^-KEAP1 Interaction by PYR

**Figure 4:**
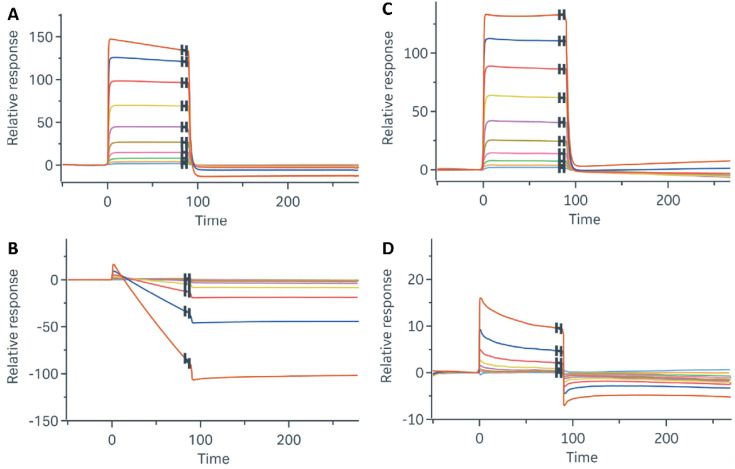
PYR modestly enhances DLGW24C–Kelch interaction in SPR assays. SPR sensorgrams showing binding of (**A**) DLG^WT^ peptide and (**B**) DLG^W24C^ peptide to the KEAP1 Kelch domain. (**C**) PYR has minimal effect on DLG^WT^–Kelch interaction. (**D**) PYR modestly increases binding of DLG^W24C^ peptide to the Kelch domain. The colors in an SPR sensorgrams distinguish different concentrations of the analyte (DLG peptide).

To assess whether PYR influences the interaction between NRF2 and KEAP1 at the biophysical level, SPR assays were performed using DLG-containing NRF2 peptides and a Kelch domain fragment of KEAP1. As expected, the DLG^WT^ peptide bound the Kelch domain, whereas the DLG^W24C^ peptide showed minimal binding (Figure 4A,B). Addition of PYR modestly increased the interaction between DLG^W24C^ and the Kelch domain (Figure 4C,D). ITC analysis further showed that PYR binds recombinant KEAP1 with moderate affinity (Kd = 13 µM; Figure S5). Together, these data are consistent with a glue-like facilitation of NRF2^W24C^-KEAP1 association.

**Figure 5:**
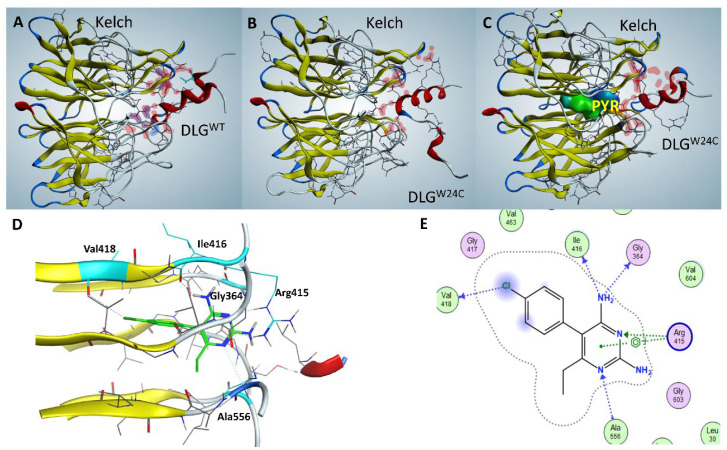
Computational modeling suggests PYR may facilitate NRF2^W24C^–KEAP1 interaction via the Kelch domain. (**A**) Predicted interaction interface between the DLG^WT^ motif and the Kelch domain. (**B**) Predicted weakening of the DLG^W24C^–Kelch interaction. (**C**) Docking of PYR into a Kelch domain pocket increases predicted interaction surface area and shape complementarity for the DLG^W24C^–Kelch complex. (**D**) Close-up view of PYR positioned within the Kelch domain cavity. (**E**) Two-dimensional interaction map showing predicted contacts between PYR and Kelch domain residues.

Computational docking analyses identified several potential PYR interaction sites on KEAP1, including a pocket within the Kelch domain that exhibited the highest confidence scores and favorable geometry for ternary complex formation (Figure S6). Docking simulations indicated that PYR binding increased the predicted interaction surface area and improved shape complementarity between NRF2^W24C^ and the Kelch domain (Figure 5A–C). Residues predicted to interact with PYR included Arg^415^, Ala^556^, Gly^364^, Ile^416^, and Val^418^ (Figure 5D,E). However, due to the limited resolution of computational docking, it was not possible to reliably distinguish between different binding modes of PYR across the various NRF2^Mut^–KEAP1 complexes. As such, the docking results should be interpreted cautiously and are insufficient to definitively resolve mutation-specific interaction differences.

In contrast to NRF2 activators that disrupt NRF2-KEAP1 binding by occupying the canonical DLG interface and engaging Arg^415^ directly [20,21,22] (Figure S7), PYR was predicted to bind an adjacent pocket within the Kelch domain. This distinct binding mode may favor NRF2–KEAP1 association rather than disruption. Independent validation using Chemistry42 and MDflow simulations supported the stability of this predicted interaction mode (Figure S8).

## 4. Discussion

This study provides new insights into the functional consequences of NRF2 activation in ESCC and highlights PYR as an NRF2 inhibitor with therapeutic relevance. Using genetically defined isogenic ESCC cell lines with targeted disruption of NRF2 or KEAP1, we demonstrated that NRF2 activation produces broadly similar but context-dependent effects on cellular behaviors, chemosensitivity, and radiosensitivity, depending on the underlying genetic background. A central finding of this work is that PYR promotes KEAP1-dependent degradation of NRF2^W24C^ within a few hours, while other NRF2^Mut^ show limited or no response (Figure 3 and Figure S4). This effect is associated with enhanced NRF2^W24C^-KEAP1 interaction rather than a high-affinity binding mechanism.

Our biochemical and cellular data suggest that this degradation effect depends on KEAP1 and is associated with altered engagement of the Kelch domain. Molecular docking analyses predict that PYR may interact with a pocket within the Kelch domain, thereby stabilizing the otherwise weakened interaction between NRF2^W24C^ and KEAP1. Functionally, this enhanced interaction correlates with increased NRF2 ubiquitination and proteasomal degradation, leading to suppression of NRF2 signaling and restoration of sensitivity to chemotherapy and radiation. From a therapeutic perspective, these findings suggest that pharmacologic reinforcement of KEAP1-mediated NRF2 turnover may represent a viable strategy to overcome NRF2-driven treatment resistance in ESCC, particularly given that the PYR concentrations used in this study (10–20 µM) are physiologically relevant [8].

Previously reported NRF2 degraders, such as VVD-130037 and VVD-065, operate through a distinct mechanism by targeting the BTB domain of KEAP1 and promoting KEAP1-CUL3 interactions [13]. In contrast, PYR appears to influence NRF2 stability through modulation of the Kelch domain, potentially offering a mechanistically distinct and complementary approach. Notably, VVD-065 has been shown to induce bidirectional conformational changes in the BTB domain that differ from those elicited by NRF2 activators such as CDDO compounds, despite targeting the same cysteine residue, Cys^151^ [26]. This bidirectional sensing mechanism allows KEAP1 to respond to both activating and inhibitory chemical cues. Analogously, our docking analyses suggest that PYR and several NRF2 activators may engage overlapping residues within the Kelch domain, including Arg^415^ (Figure 5 and Figure S7), yet produce opposite functional outcomes. Such observations raise the possibility that, similar to the BTB domain, the Kelch domain may act as a tunable interface that fine-tunes NRF2-KEAP1 affinity and downstream NRF2 activity.

While our biochemical and biophysical analyses support a glue-like activity underlying PYR’s mechanism of action, several important limitations should be acknowledged. First, the observed biophysical interaction between PYR, KEAP1, and NRF2^W24C^ is relatively modest and may involve multiple low-affinity contacts rather than a single, well-defined high-affinity interface. Consistent with this, ITC analyses suggest a binding stoichiometry indicative of multiple interaction sites, potentially including allosteric, surface-exposed, or cysteine-proximal regions in addition to the predicted Kelch pocket. Accordingly, PYR should not be considered a specific molecular glue targeting a single defined site. Furthermore, our computational modeling is entirely in silico and remains hypothetical, limiting confidence in the proposed binding modes without experimental structural validation.

Additional limitations arise from the cellular systems used in this study. Our analysis relies on a limited number of isogenic models, primarily KYSE70 and KYSE450 cells, and include only two mutants (W24C and D77V), restricting the generalizability of our findings. Broader validation across additional cell lines will be necessary. Moreover, although NRF2^null^-KYSE70 was employed, NRF2 expression was not fully abolished in these cells. Without complementary suppression strategies or rescue/reciprocal expression experiments, our mechanistic interpretation is limited. It also remains possible that PYR affects NRF2 stability indirectly through off-target mechanisms. At the functional level, our study primarily focused on NRF2 protein abundance and did not systematically assess downstream NRF2 target gene expression or pathway activity. In addition, comprehensive dose–response and time-course analyses are lacking, which may obscure important dynamics of NRF2 degradation. Finally, key hallmarks of molecular glue activity were not fully established for PYR, including quantitative characterization of ternary complex formation, demonstration of a clear relationship between ternary complex stoichiometry and target degradation, and mutational disruption of the predicted small-molecule binding interface leading to loss of both binding and degradation. Addressing these limitations through expanded cellular models, orthogonal perturbation strategies, and high-resolution structural and quantitative biochemical studies will be essential to more precisely defining the NRF2-degrading mechanism of PYR in future studies.

It is important to emphasize that inhibition of DHFR remains the primary mechanism underlying PYR’s broader NRF2-inhibitory activity [16], while KEAP1-dependent degradation may only contributes to early or mutation-specific NRF2 suppression. Through suppression of folate-dependent one-carbon metabolism, PYR can attenuate NRF2 expression independently of KEAP1 and has also been shown to inhibit STAT3 signaling [27]. Consistent with this mechanism, PYR reduced STAT3 target gene expression in chronic lymphocytic leukemia patients who exhibited favorable clinical responses [28]. However, systemic toxicity associated with DHFR inhibition [29], together with PYR’s effects on both NRF2^Mut^ and NRF2^WT^ at longer treatment durations (Figure 3E,F), likely constrains its therapeutic window. Nonetheless, the identification of a potential Kelch interaction site highlights an opportunity to develop next-generation KEAP1-targeting agents that retain mutation-selective NRF2 suppression while minimizing DHFR-related liabilities. Beyond NRF2 and STAT3, PYR has been reported to reduce protein levels of AIMP2-DX2 without affecting mRNA expression [17], and to inhibit additional oncogenic pathways, including NFκB, MAPK, thymidine phosphorylase, and telomerase [30]. Since most of these data are correlative and indirect, it remains unclear whether these effects reflect direct binding or indirect metabolic consequences.

Another notable observation from this study is that NRF2^WT^ and NRF2^Mut^ exert distinct effects on ESCC cell behavior. Overexpression of NRF2^WT^ in KEAP1^null^-KYSE450 cells promoted squamous differentiation and suppressed proliferation, whereas expression of NRF2^W24C^ produced mixed effects on differentiation markers and enhanced proliferation in KYSE70 cells (Figure 1). These findings align with in vivo studies showing that combined expression of NRF2^L30F^ and TP53^R172H^ induces ESCC-like lesions, whereas KEAP1 loss in the same context fails to do so and results in loss of esophageal epithelial cells over time [31]. Similarly, NRF2^WT^ overexpression suppresses proliferation in several ESCC models, while NRF2^Mut^ such as NRF2^R34Q^ and NRF2^E79K^ promote tumor cell growth [32]. Together, these observations support a model in which NRF2^WT^ may function as a context-dependent tumor suppressor in ESCC, whereas NRF2^Mut^ acts as an oncogenic driver, underscoring the need for mutation-selective NRF2-targeting strategies rather than broad NRF2 inhibition. However, this distinction should not be considered a universal rule, as our conclusions are derived from a limited set of cellular models and are informed in part by prior in vivo evidence. Further validation across additional systems will be required to establish the generalizability of this framework.

Although *Keap1* knockout phenotypes in the esophagus have largely been attributed to NRF2 hyperactivation [31], it remains possible that NRF2-independent KEAP1 substrates also contribute to the observed phenotypes. Indeed, genetic activation of NRF2 in *Keap1^-/-^* mice leads to esophageal hyperplasia and hyperkeratosis, phenotypes that are fully rescued by concurrent *Nrf2* deletion [33,34]. Nevertheless, KEAP1 has been reported to interact with multiple proteins beyond NRF2 [35], raising the possibility that additional KEAP1-regulated pathways may influence epithelial homeostasis and tumorigenesis.

## 5. Conclusions

Targeting the KEAP1–NRF2 axis is challenging due to the context-dependent role of NRF2. Genetic heterogeneity, including NRF2 and KEAP1 mutations, further limits the effectiveness of a single therapeutic strategy. In addition, the KEAP1–NRF2 protein–protein interaction is difficult to drug, and potential off-target effects, compensatory pathways, and pharmacokinetic limitations may reduce efficacy and safety. This study expands our understanding of PYR as a dual-mode inhibitor of NRF2 signaling with mutation-selective therapeutic potential. By combining DHFR-dependent metabolic suppression of NRF2 expression with a KEAP1-dependent degradation effect on NRF2^Mut^, PYR functionally restores NRF2 control and sensitizes ESCC cells to chemotherapy and radiation. These findings support NRF2 mutation status as a predictive biomarker and provide a rationale for developing refined KEAP1-targeting strategies to selectively treat NRF2^Mut^-driven cancers including ESCC.

## Data Availability

The data generated in this study are available upon request from the corresponding author.

## Supplementary Materials

The following supporting information can be downloaded at https://www.mdpi.com/article/10.3390/cancers18091354/s1, Figure S1. Generation and validation of NRF2^null^-KYSE70 cells; Figure S2. Generation and validation of KEAP1^null^-KYSE450 and KEAP1^null^-KYSE70 cells; Figure S3. NRF2 modulates chemosensitivity and radiosensitivity of ESCC cells; Figure S4. Mutation-specific effects of PYR on NRF2 protein stability; Figure S5. ITC analysis of PYR binding to KEAP1; Figure S6. Computational prediction of PYR-KEAP1 interaction sites; Figure S7. Docking of NRF2-activating compounds that disrupt NRF2-KEAP1 interaction; Figure S8. Chemistry42 and MDflow simulations of PYR-KEAP1 interaction; Table S1. Antibody information; Excel S1. RNAseq data for differential gene expression in KYSE70 cells: NRF2^null^-KYSE70 vs. parental NRF2^W24C^-KYSE70 cells; Excel S2: RNAseq data for differential gene expression in KYSE450 cells: KEAP1^null^-KYSE450 vs. parental NRF2^WT^-KYSE450 cells; Excel S3. Metabolomics analysis of KYSE70 cells: NRF2^null^-KYSE70 vs. parental NRF2^W24C^-KYSE70 cells. File S1. Original western blots.

## Abbreviations

The following abbreviations are used in this manuscript:

AIMP2-DX2	exon 2-depleted splice variant of aminoacyl-tRNA synthetase-interacting multifunctional protein 2
CI	combination index
DEG	differentially expressed gene
Kd	dissociation constant
ESCC	esophageal squamous cell carcinoma
5-FU	5-fluorouracil
IP	immunoprecipitation
ITC	isothermal titration calorimetry
KEAP1	Kelch-like ECH-associated protein 1
MTX	methotrexate
Mut	mutant
NRF2/NFE2L2	nuclear factor erythroid 2-related factor 2
PLA	proximity ligation assay
PYR	pyrimethamine
SPR	surface plasmon resonance
WT	wild-type

## References

[1] Kang X., Chen K., Li Y., Li J., D’Amico T.A., Chen X. (2015). Personalized targeted therapy for esophageal squamous cell carcinoma. World J. Gastroenterol..

[2] Shah M.A., Altorki N., Patel P., Harrison S., Bass A., Abrams J.A. (2023). Improving outcomes in patients with oesophageal cancer. Nat. Rev. Clin. Oncol..

[3] Liu Z., Zhao Y., Kong P., Liu Y., Huang J., Xu E., Wei W., Li G., Cheng X., Xue L. (2023). Integrated multi-omics profiling yields a clinically relevant molecular classification for esophageal squamous cell carcinoma. Cancer Cell.

[4] Ma S., Paiboonrungruan C., Yan T., Williams K.P., Major M.B., Chen X.L. (2018). Targeted therapy of esophageal squamous cell carcinoma: The NRF2 signaling pathway as target. Ann. N. Y. Acad. Sci..

[5] Rojo de la Vega M., Chapman E., Zhang D.D. (2018). NRF2 and the Hallmarks of Cancer. Cancer Cell.

[6] Hirose W., Oshikiri H., Taguchi K., Yamamoto M. (2022). The KEAP1-NRF2 System and Esophageal Cancer. Cancers.

[7] Iwasaki T., Shirota H., Sasaki K., Ouchi K., Nakayama Y., Oshikiri H., Otsuki A., Suzuki T., Yamamoto M., Ishioka C. (2024). Specific cancer types and prognosis in patients with variations in the KEAP1-NRF2 system: A retrospective cohort study. Cancer Sci..

[8] Li Y., Ladd Z., Xiong Z., Bui-Linh C., Paiboonrungruang C., Subramaniyan B., Li H., Wang H., Balch C., Shersher D.D. (2025). Lymphatic Metastasis of Esophageal Squamous Cell Carcinoma: The Role of NRF2 and Therapeutic Strategies. Cancers.

[9] Kerins M.J., Ooi A. (2018). A catalogue of somatic NRF2 gain-of-function mutations in cancer. Sci. Rep..

[10] Chen F., Xiao M., Hu S., Wang M. (2024). Keap1-Nrf2 pathway: A key mechanism in the occurrence and development of cancer. Front. Oncol..

[11] Bruschweiler S., Fuchs J.E., Bader G., McConnell D.B., Konrat R., Mayer M. (2021). A Step toward NRF2-DNA Interaction Inhibitors by Fragment-Based NMR Methods. ChemMedChem.

[12] Bushweller J.H. (2019). Targeting transcription factors in cancer—From undruggable to reality. Nat. Rev. Cancer.

[13] Roy N., Wyseure T., Lo I.C., Lu J., Eissler C.L., Bernard S.M., Bok I., Snead A.N., Parker A., Lo U.G. (2025). A covalent allosteric molecular glue suppresses NRF2-dependent cancer growth. Cancer Discov..

[14] Kuhlmann F.W., Fleckenstein J.M., Cohen J., Powderly W.G., Opal S.M. (2017). Antiparasitic Agents. Infectious Diseases.

[15] Paiboonrungruang C., Xiong Z., Lamson D., Li Y., Bowman B., Chembo J., Huang C., Li J., Livingston E.W., Frank J.E. (2023). Small molecule screen identifies pyrimethamine as an inhibitor of NRF2-driven esophageal hyperplasia. Redox Biol..

[16] Chembo J., Bowman B.M., Lapak K., Wilkerson E., Wamsley N.T., Paiboonrungruang C., Cho K., Medcalf M.R., Wang H., Patti G.J. (2025). Pyrimethamine and a potent analog inhibit NRF2 by suppressing one-carbon metabolism. J. Biol. Chem..

[17] Kim D.G., Park C.M., Huddar S., Lim S., Kim S., Lee S. (2020). Anticancer Activity of Pyrimethamine via Ubiquitin Mediated Degradation of AIMP2-DX2. Molecules.

[18] Mikac S., Dziadosz A., Padariya M., Kalathiya U., Fahraeus R., Marek-Trzonkowska N., Chrusciel E., Urban-Wojciuk Z., Papak I., Arcimowicz L. (2022). Keap1-resistant ΔN-Nrf2 isoform does not translocate to the nucleus upon electrophilic stress. bioRxiv.

[19] Fu J., Xiong Z., Huang C., Li J., Yang W., Han Y., Paiboonrungruan C., Major M.B., Chen K.N., Kang X. (2019). Hyperactivity of the transcription factor Nrf2 causes metabolic reprogramming in mouse esophagus. J. Biol. Chem..

[20] Dayalan Naidu S., Suzuki T., Dikovskaya D., Knatko E.V., Higgins M., Sato M., Novak M., Villegas J.A., Moore T.W., Yamamoto M. (2022). The isoquinoline PRL-295 increases the thermostability of Keap1 and disrupts its interaction with Nrf2. iScience.

[21] Davies T.G., Wixted W.E., Coyle J.E., Griffiths-Jones C., Hearn K., McMenamin R., Norton D., Rich S.J., Richardson C., Saxty G. (2016). Monoacidic Inhibitors of the Kelch-like ECH-Associated Protein 1: Nuclear Factor Erythroid 2-Related Factor 2 (KEAP1:NRF2) Protein-Protein Interaction with High Cell Potency Identified by Fragment-Based Discovery. J. Med. Chem..

[22] Asano W., Hantani R., Uhara T., Debaene F., Nomura A., Yamaguchi K., Adachi T., Otake K., Harada K., Hantani Y. (2024). Screening approaches for the identification of Nrf2-Keap1 protein-protein interaction inhibitors targeting hot spot residues. SLAS Discov..

[23] Ivanenkov Y.A., Polykovskiy D., Bezrukov D., Zagribelnyy B., Aladinskiy V., Kamya P., Aliper A., Ren F., Zhavoronkov A. (2023). Chemistry42: An AI-Driven Platform for Molecular Design and Optimization. J. Chem. Inf. Model..

[24] Kopacz A., Rojo A.I., Patibandla C., Lastra-Martinez D., Piechota-Polanczyk A., Kloska D., Jozkowicz A., Sutherland C., Cuadrado A., Grochot-Przeczek A. (2022). Overlooked and valuable facts to know in the NRF2/KEAP1 field. Free Radic. Biol. Med..

[25] Sun Z., Zhang S., Chan J.Y., Zhang D.D. (2007). Keap1 controls postinduction repression of the Nrf2-mediated antioxidant response by escorting nuclear export of Nrf2. Mol. Cell Biol..

[26] Suzuki T., Takagi K., Iso T., Wen H., Zhang A., Hatakeyama T., Oshima H., Mizushima T., Yamamoto M. (2025). Bidirectional regulation of KEAP1 BTB domain-based sensor activity. Redox Biol..

[27] Heppler L.N., Attarha S., Persaud R., Brown J.I., Wang P., Petrova B., Tosic I., Burton F.B., Flamand Y., Walker S.R. (2022). The antimicrobial drug pyrimethamine inhibits STAT3 transcriptional activity by targeting the enzyme dihydrofolate reductase. J. Biol. Chem..

[28] Brown J.R., Walker S.R., Heppler L.N., Tyekucheva S., Nelson E.A., Klitgaard J., Nicolais M., Kroll Y., Xiang M., Yeh J.E. (2021). Targeting constitutively active STAT3 in chronic lymphocytic leukemia: A clinical trial of the STAT3 inhibitor pyrimethamine with pharmacodynamic analyses. Am. J. Hematol..

[29] Ben-Harari R.R., Goodwin E., Casoy J. (2017). Adverse Event Profile of Pyrimethamine-Based Therapy in Toxoplasmosis: A Systematic Review. Drugs R. D.

[30] Ramchandani S., Mohan C.D., Mistry J.R., Su Q., Naz I., Rangappa K.S., Ahn K.S. (2022). The multifaceted antineoplastic role of pyrimethamine against human malignancies. IUBMB Life.

[31] Takahashi J., Suzuki T., Sato M., Nitta S., Yaguchi N., Muta T., Tsuchida K., Suda H., Morita M., Hamada S. (2024). Differential squamous cell fates elicited by NRF2 gain of function versus KEAP1 loss of function. Cell Rep..

[32] Cui Y., Chen H., Xi R., Cui H., Zhao Y., Xu E., Yan T., Lu X., Huang F., Kong P. (2020). Whole-genome sequencing of 508 patients identifies key molecular features associated with poor prognosis in esophageal squamous cell carcinoma. Cell Res..

[33] Suzuki T., Seki S., Hiramoto K., Naganuma E., Kobayashi E.H., Yamaoka A., Baird L., Takahashi N., Sato H., Yamamoto M. (2017). Hyperactivation of Nrf2 in early tubular development induces nephrogenic diabetes insipidus. Nat. Commun..

[34] Wakabayashi N., Itoh K., Wakabayashi J., Motohashi H., Noda S., Takahashi S., Imakado S., Kotsuji T., Otsuka F., Roop D.R. (2003). Keap1-null mutation leads to postnatal lethality due to constitutive Nrf2 activation. Nat. Genet..

[35] Kopacz A., Kloska D., Forman H.J., Jozkowicz A., Grochot-Przeczek A. (2020). Beyond repression of Nrf2: An update on Keap1. Free Radic. Biol. Med..

