# Supplementary material for: Pyrimethamine Restores KEAP1-Mediated Degradation of Select NRF2 Mutants in Esophageal Squamous Cell Carcinoma
Source: Cancers (Basel). 2026 Apr 24;18(9):1354. doi: 10.3390/cancers18091354 (PMC13163009; doi:10.3390/cancers18091354)

Figure 1A (1: KYSE70; 2: NRF2^{null}-KYSE70: 3: KYSE450; 4: KEAP1^{null}-KYSE450)

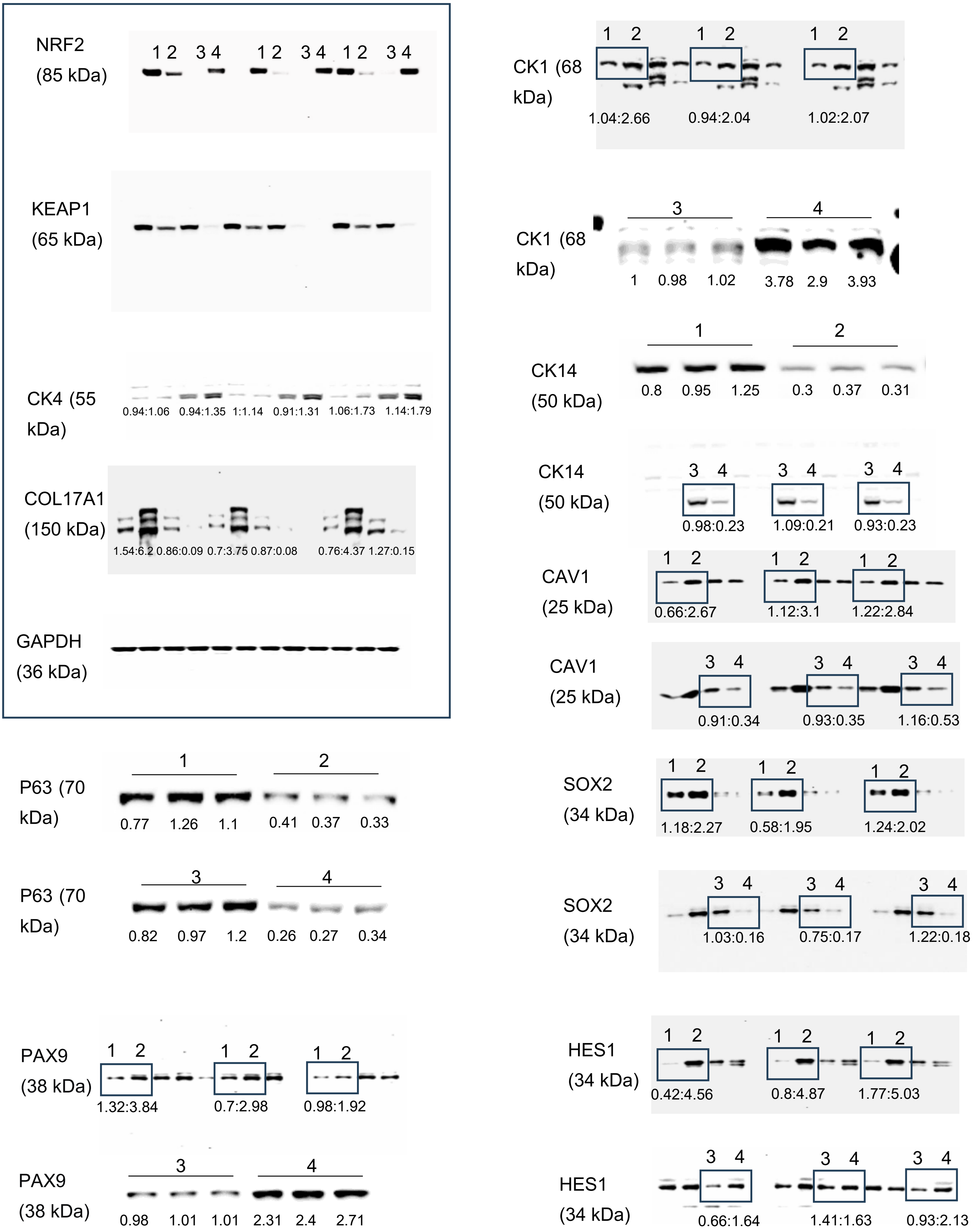

Figure 3A (KYSE70, PYR treatment)

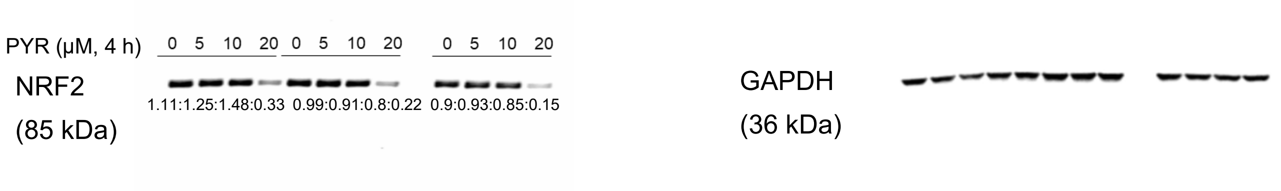

Figure 3B (KEAP1^{null}-KYSE70, PYR treatment)

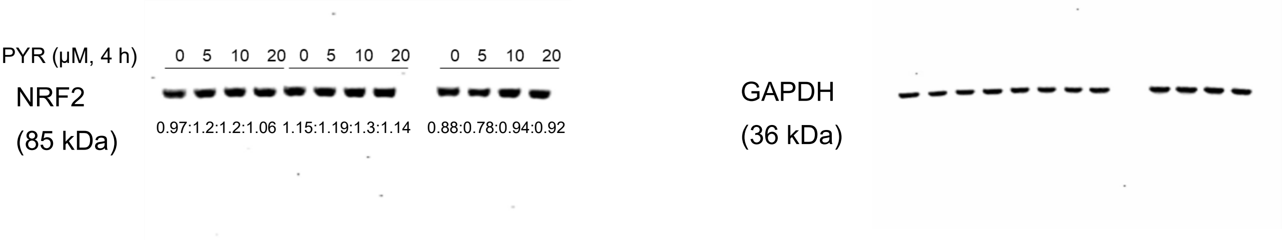

Figure 3C

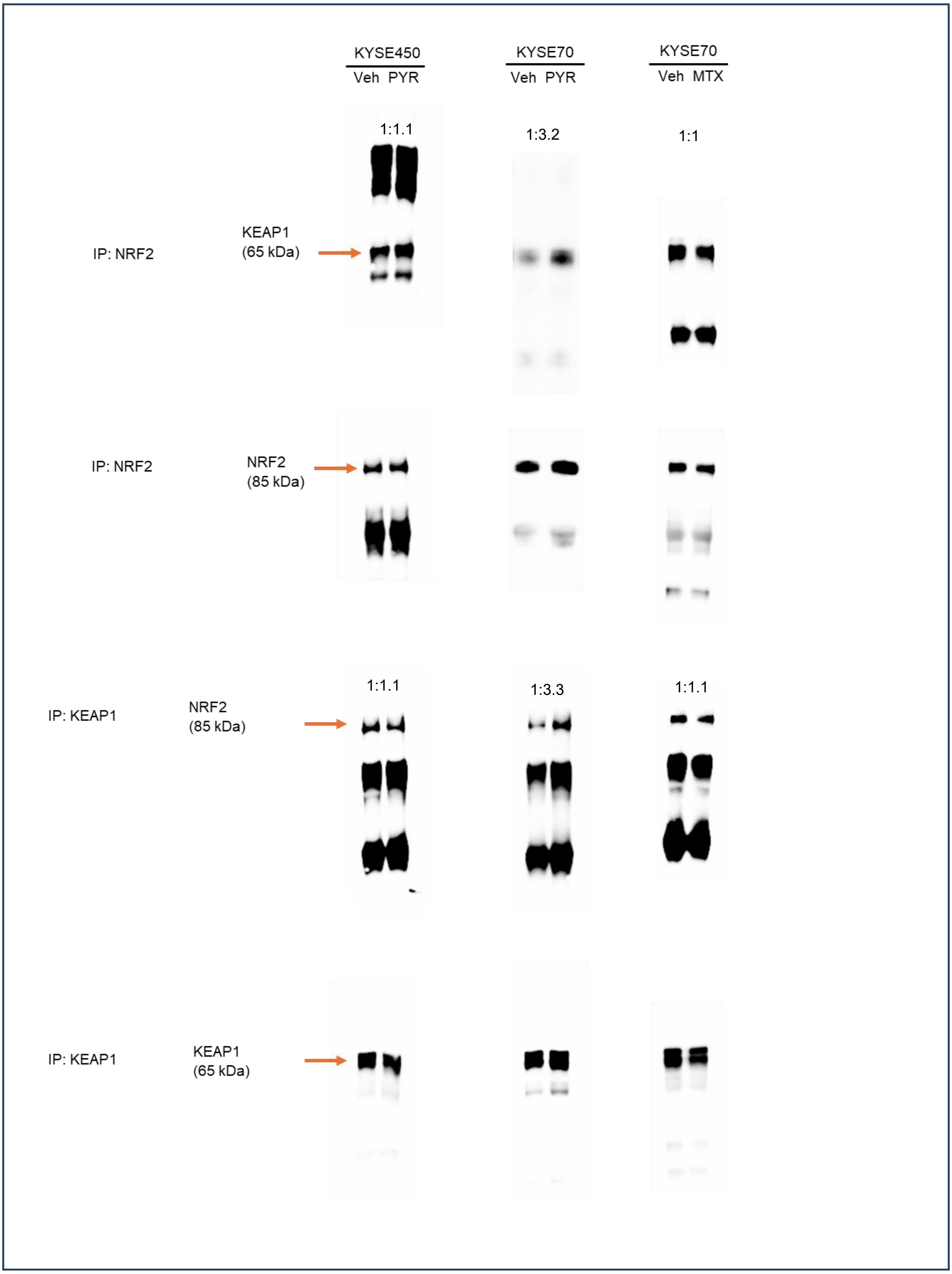
KYSE450
Veh PYR
KYSE70
Veh PYR
KYSE70
Veh MTX
1:1.1
1:3.2
1:1
IP: NRF2
KEAP1
(65 kDa)
IP: NRF2
NRF2
(85 kDa)
1:1.1
1:3.3
1:1.1
IP: KEAP1
NRF2
(85 kDa)
IP: KEAP1
KEAP1
(65 kDa)

Figure 3C (continued)

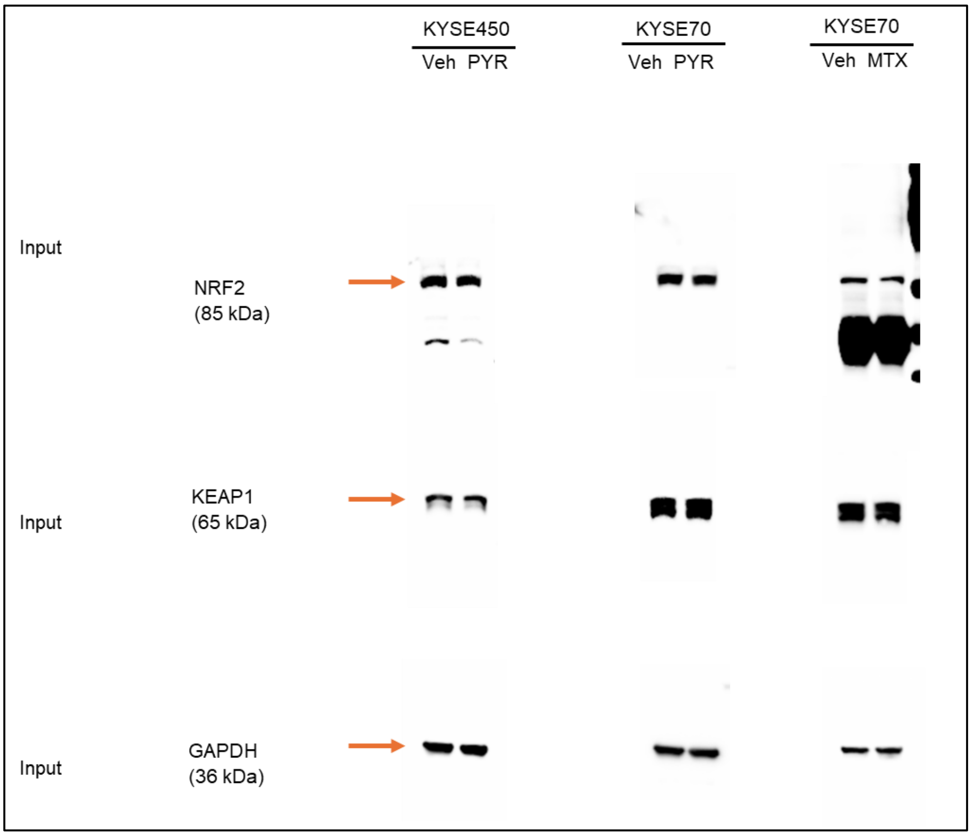

Figure 3 E

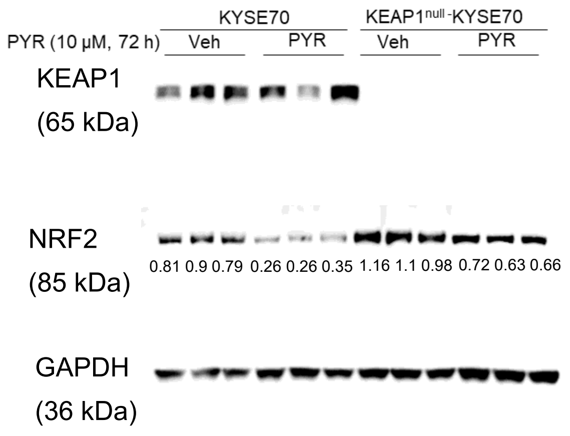

Figure 3F

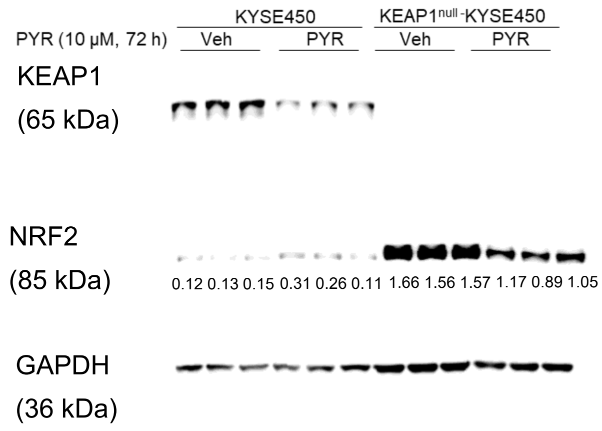

Supplementary Figure 4A (NRF2^{D77V}-KYSE180, PYR treatment)

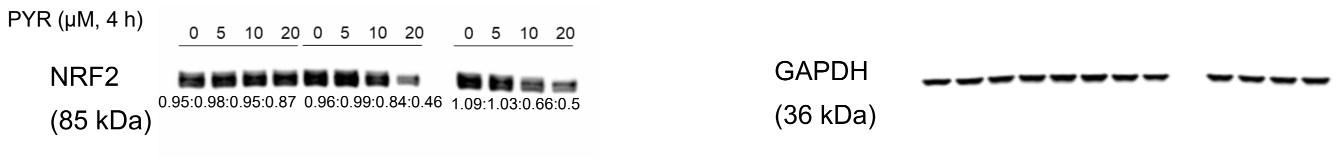

Supplementary Figure 4B (NRF2^{WT}-KYSE450, PYR treatment)

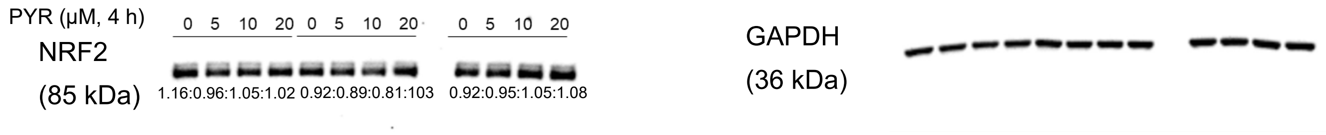

Supplementary Figure 4C (NRF2^{D29H}-TE14, PYR treatment)

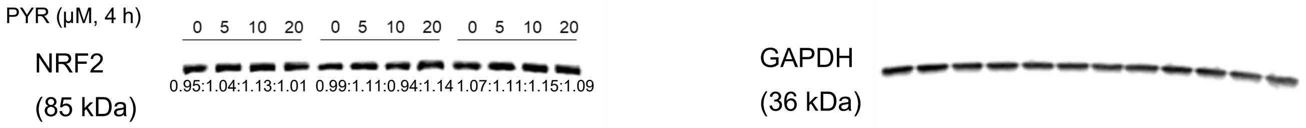

Supplementary Figure 4 D (NRF2^{G81S}-OE21, PYR treatment)

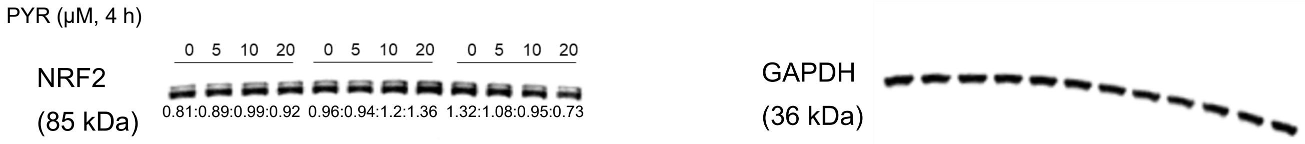

Supplementary Figure 4E (KYSE70)

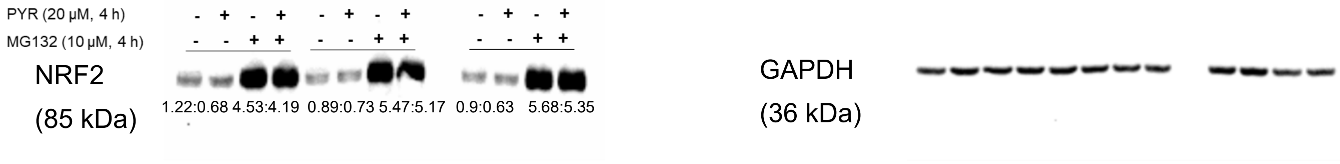

Supplement: Supplementary file 1 [file cancers-18-01354-s001.zip › File S1.pdf]
