# Supplementary material for: Pyrimethamine Restores KEAP1-Mediated Degradation of Select NRF2 Mutants in Esophageal Squamous Cell Carcinoma
Source: Cancers (Basel). 2026 Apr 24;18(9):1354. doi: 10.3390/cancers18091354 (PMC13163009; doi:10.3390/cancers18091354)
Supplement: Supplementary file 1 [file cancers-18-01354-s001.zip › supplementary figures.pdf]

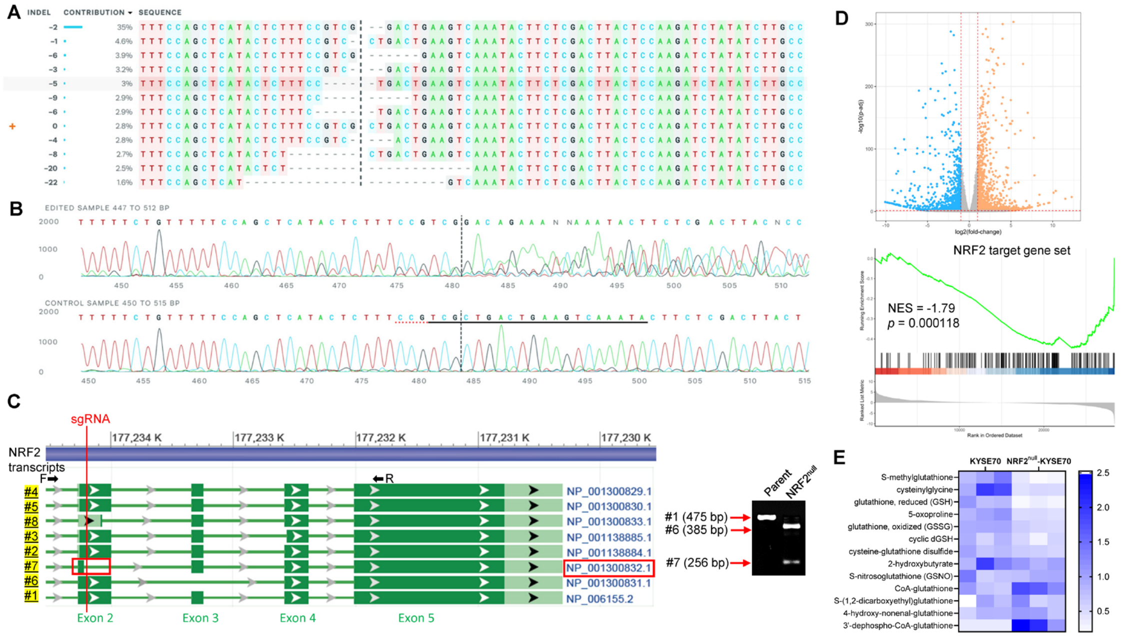

Figure S1. Generation and validation of NRF2^{null}-KYSE70 cells. (A) Among the CRISPRed clones, -2 deletion was the most abundant one (35%) and selected for monoclonal expansion. The sgRNA target sequence for NRF2 was 5′-TATTTGACTTCAGTCAGCGA-3′. (B) Sanger sequencing confirming a frameshift mutation and premature stop codon at amino acid 46 in exon 2. (C) A scheme of NRF2 transcript variants (#1-8) was generated according to NCBI. Vertical red line indicated that transcript 7 was missed by sgRNA. RT-PCR confirmed that transcript 6 (385bp) and transcript 7 (256bp) were expressed in the NRF2^{null}-KYSE70 cells. Locations of forward and reverse primers were labeled. (D) Volcano plot of RNAseq data analysis and GSEA of NRF2^{null}-KYSE70 cells in comparison to its parental NRF2^{W24C}-KYSE70 cells showed that the human NRF2 target gene set was significantly downregulated in NRF2^{null}-KYSE70 *vs* NRF2^{W24C}-KYSE70 cells. (E) Metabolomic analysis showed that glutathione metabolism was significantly suppressed in NRF2^{null}-KYSE70 *vs* NRF2^{W24C}-KYSE70 cells.

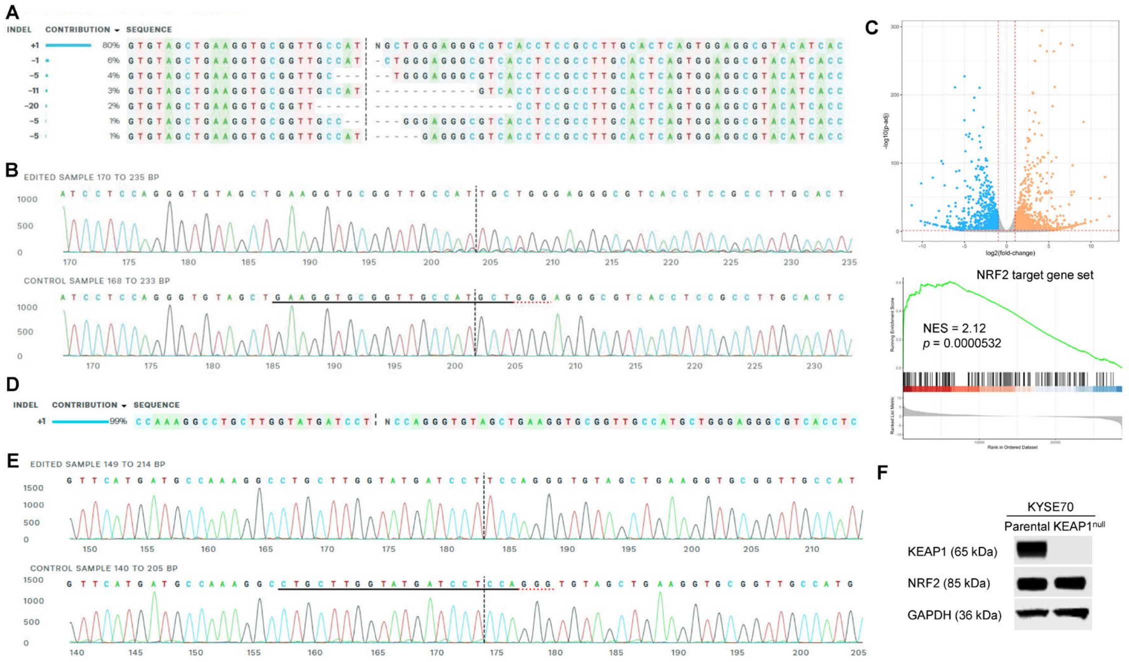

Figure S2. Generation and validation of KEAP1^{null}-KYSE450 and KEAP1^{null}-KYSE70 cells. (A) Among the CRISPRed clones of KYSE450 cells, +1 insertion was the most abundant one (80%) and chosen by monoclonal selection for subsequent experiments. The sgRNA target sequence for KEAP1 was 5′-GAAGGTGCGGTTGCCATGCT-3′. (B) Sanger sequencing validated +1 insertion in exon 2 which was expected to result in homozygous KEAP1 knockout. (C) Volcano plot of RNAseq data analysis and GSEA of KEAP1^{null}-KYSE450 cells in comparison with its parental NRF2^{WT}-KYSE450 cells showed that the human NRF2 target gene set was significantly upregulated in KEAP1^{null}-KYSE450 cells *vs* NRF2^{WT}-KYSE450 cells. (D) Among the CRISPRed clones of KYSE70 cells, +1 insertion was the most abundant one (99%) and chosen by monoclonal selection for subsequent experiments. The sgRNA target sequence for KEAP1 was 5′-CTGCTTGGTATGATCCTCCA-3′. (E) Sanger sequencing validated +1 insertion in exon 2 which is expected to result in homozygous KEAP1 knockout. (F) KEAP1^{null}-KYSE70 cells were validated by absence of KEAP1 expression on Western blot.

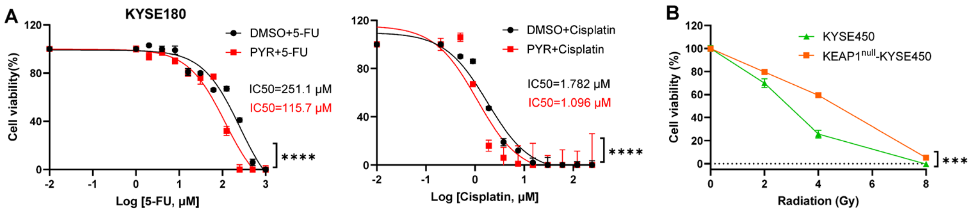

Figure S3. NRF2 modulates chemosensitivity and radiosensitivity of ESCC cells. (A) Viability of NRF2^{D77V}-KYSE180 cells after co-treatment with PYR (10 μM) and 5-FU or cisplatin; (B) Viability of NRF2^{WT}-KYSE450 and KEAP1^{null}-KYSE450 cells in 3D culture when exposed to radiation. Data are shown as mean ± SD. *** $p < 0.001$; **** $p < 0.0001$.

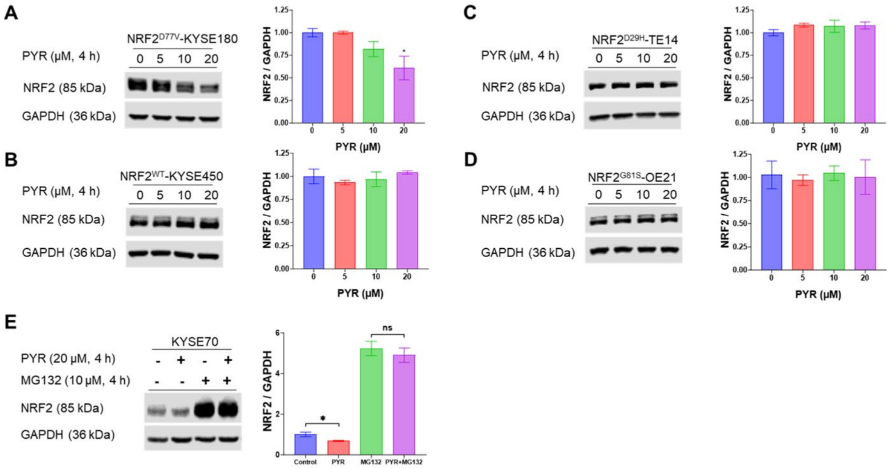

Figure S4. Mutation-specific effects of PYR on NRF2 protein stability. (A–D) NRF2 protein levels following 4 h PYR treatment (10 μM) in ESCC cells expressing different NRF2 variants. (E) Proteasome inhibition with MG132 abolishes PYR-induced NRF2^{W24C} degradation. Data are shown as mean ± SD. * $p < 0.05$.

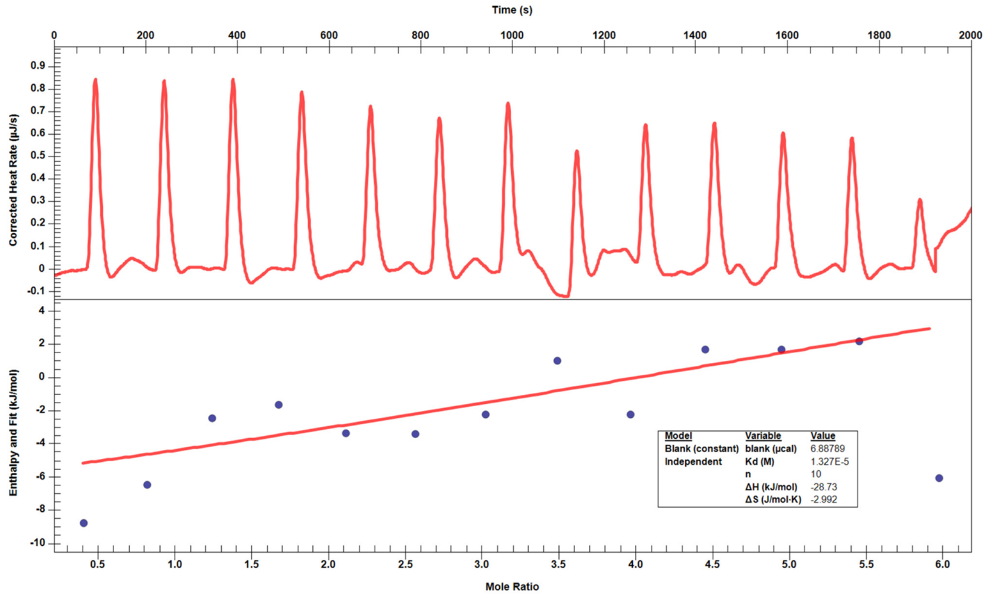

Figure S5. ITC analysis of PYR binding to KEAP1. Representative ITC binding curve for PYR and recombinant human KEAP1.

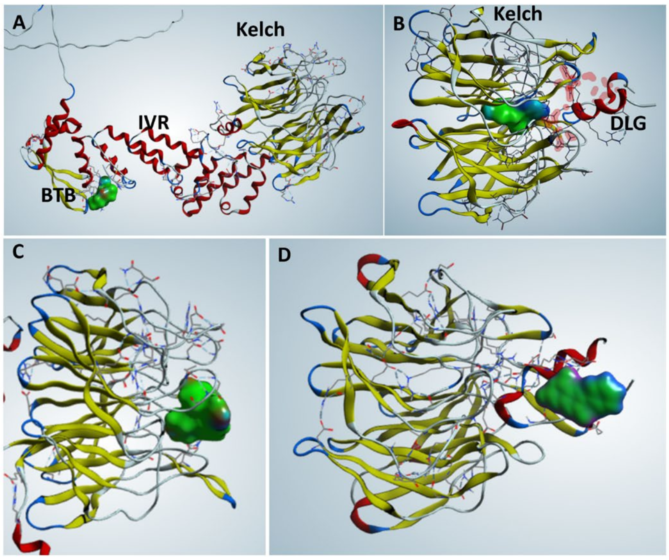

Figure S6. Computational prediction of PYR-KEAP1 interaction sites. DiffDock-identified candidate PYR interaction sites on KEAP1, including BTB (A), Kelch pocket (B), surface pocket (C), and DLG-binding regions (D).

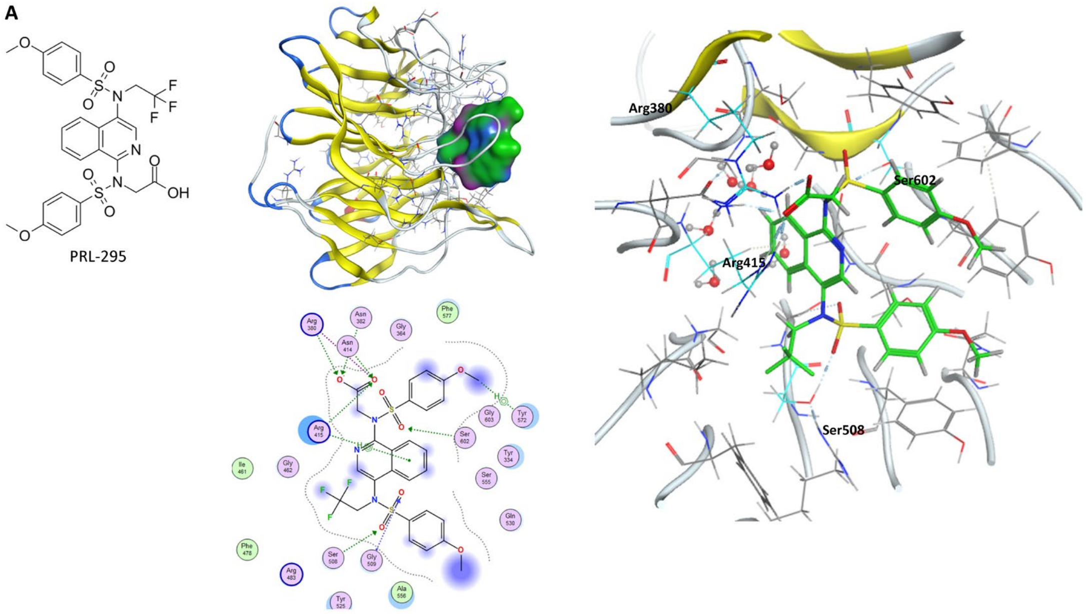
A
PRL-295
Arg380
Ser602
Arg415
Ser508

(a)

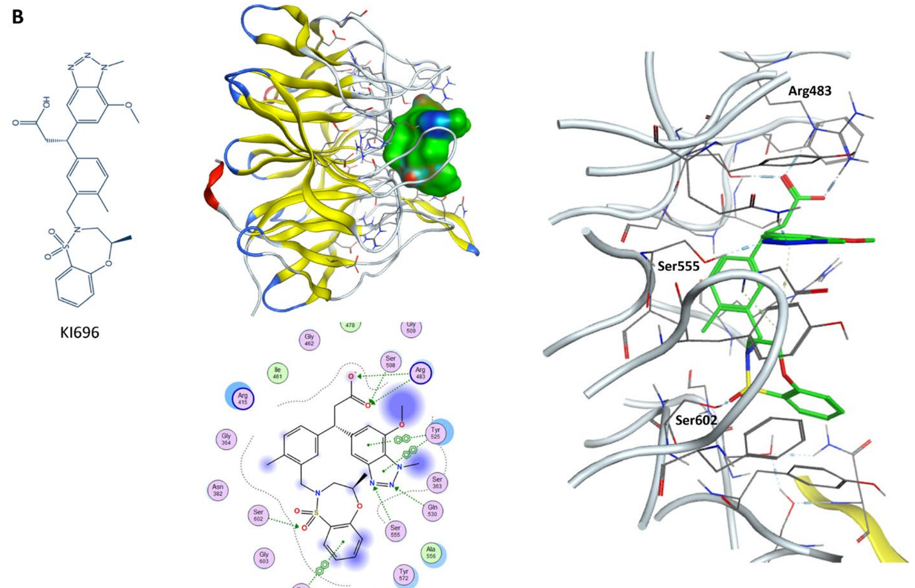
B
KI696
Arg483
Ser555
Ser602

(b)

C

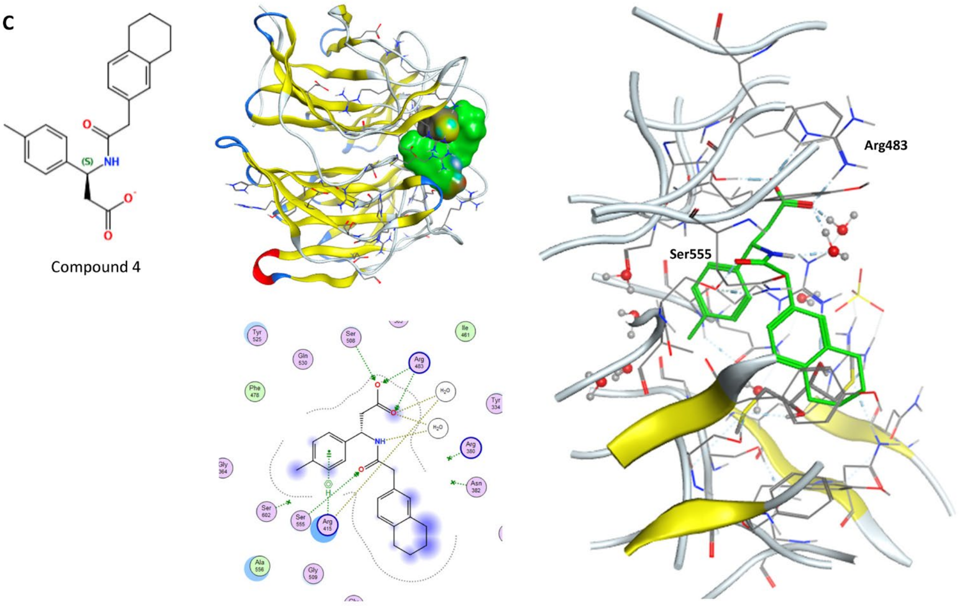

(c)

Figure S7. Docking of NRF2-activating compounds that disrupt NRF2-KEAP1 interaction. Predicted binding modes of PRL-295 (A), KI696 (B), and Compound 4 (C) within the Kelch domain.

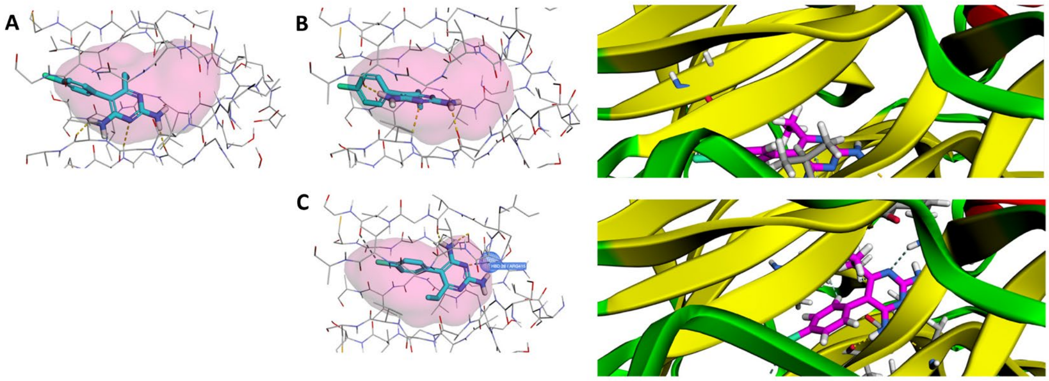

Figure S8. Chemistry42 and MDflow simulations of PYR-KEAP1 interaction. (A) Predicted PYR-binding pocket on KEAP1. (B) Without any constraints, the protein-ligand interaction score was calculated as -7.7. MDflow molecular dynamics simulations suggest the most plausible binding mode. (C) When a mandatory point was placed on Arg^{415}, the protein-ligand interaction score was calculated as -7.53, indicating stronger binding.
