# Supplementary material for: Pyrimethamine Restores KEAP1-Mediated Degradation of Select NRF2 Mutants in Esophageal Squamous Cell Carcinoma
Source: Cancers (Basel). 2026 Apr 24;18(9):1354. doi: 10.3390/cancers18091354 (PMC13163009; doi:10.3390/cancers18091354)
Supplement: Supplementary file 1 [file cancers-18-01354-s001.zip › Table S1.pdf]

Supplementary Table S1. Antibody information

Antigen	WB	PLA	Co-IP	Catalog No.	Host	mAb/pAb	Company	City, State
NRF2	1:1,000			20733	Rabbit	mAb	Cell Signaling	Danvers, MA
		1:200		16396-1-AP	Rabbit	pAb	Proteintech	Rosemont, IL
			-	GTX103322	Rabbit	pAb	GeneTex	Irvine, CA
KEAP1	1:2,000			ab119403	Mouse	mAb	Abcam	Waltham, MA
		1:200	-	ab119403	Mouse	mAb	Abcam	Waltham, MA
Cytokeratin 1 (CK1)	1:2,000			LS-B7799-50	Rabbit	pAb	Lifespan Biosciences	Seattle, WA
Cytokeratin 4 (CK4)	1:2,000			LS-C165630-400	Rabbit	pAb	Lifespan Biosciences	Seattle, WA
Cytokeratin 14 (CK14)	1:2,000			ab7800	Mouse	mAb	Abcam	Waltham, MA
CAV1	1:3,000			A19006	Rabbit	mAb	ABclonal	Woburn, MA
COL17A1	1:3,000			A4808	Rabbit	mAb	ABclonal	Woburn, MA
SOX2	1:2,000			ab97959	Rabbit	pAb	Abcam	Waltham, MA
P63	1:2,000			ab124762	Rabbit	mAb	Abcam	Waltham, MA
PAX9	1:2,000			12847	Rabbit	mAb	Cell Signaling	Danvers, MA
HES1	1:3,000			11988	Rabbit	mAb	Cell Signaling	Danvers, MA
GAPDH	1:20,000			ab8245	Mouse	mAb	Abcam	Waltham, MA
